# The biological applications of DNA nanomaterials: current challenges and future directions

**DOI:** 10.1038/s41392-021-00727-9

**Published:** 2021-10-08

**Authors:** Wenjuan Ma, Yuxi Zhan, Yuxin Zhang, Chenchen Mao, Xueping Xie, Yunfeng Lin

**Affiliations:** 1 State Key Laboratory of Oral Diseases, National Clinical Research Center for Oral Diseases, West China Hospital of Stomatology, Sichuan University, Chengdu, People’s Republic of China; 2 College of Biomedical Engineering, Sichuan University, Chengdu, People’s Republic of China

**Keywords:** Nanobiotechnology, Biomaterials

## Abstract

DNA, a genetic material, has been employed in different scientific directions for various biological applications as driven by DNA nanotechnology in the past decades, including tissue regeneration, disease prevention, inflammation inhibition, bioimaging, biosensing, diagnosis, antitumor drug delivery, and therapeutics. With the rapid progress in DNA nanotechnology, multitudinous DNA nanomaterials have been designed with different shape and size based on the classic Watson–Crick base-pairing for molecular self-assembly. Some DNA materials could functionally change cell biological behaviors, such as cell migration, cell proliferation, cell differentiation, autophagy, and anti-inflammatory effects. Some single-stranded DNAs (ssDNAs) or RNAs with secondary structures via self-pairing, named aptamer, possess the ability of targeting, which are selected by systematic evolution of ligands by exponential enrichment (SELEX) and applied for tumor targeted diagnosis and treatment. Some DNA nanomaterials with three-dimensional (3D) nanostructures and stable structures are investigated as drug carrier systems to delivery multiple antitumor medicine or gene therapeutic agents. While the functional DNA nanostructures have promoted the development of the DNA nanotechnology with innovative designs and preparation strategies, and also proved with great potential in the biological and medical use, there is still a long way to go for the eventual application of DNA materials in real life. Here in this review, we conducted a comprehensive survey of the structural development history of various DNA nanomaterials, introduced the principles of different DNA nanomaterials, summarized their biological applications in different fields, and discussed the current challenges and further directions that could help to achieve their applications in the future.

## Introduction

DNA was used for the storage of genetic information as a common biomolecule and fundamental hereditary material in different creatures.^1,2^ DNA materials possess the potential for nanofabrication due to its characteristic nature, i.e., high precision of Watson–Crick base-pairing and high controllability.^1,3–5^ In 1980s, Seeman et al.^4^ proposed the rules for the synthesis of DNA nanostructures for the first time, which triggered a booming years of DNA nanotechnology development.^6–10^ In the following decades, the technology of DNA nanostructures developed and improved rapidly, and various DNA nanostructures have been designed and widely applied as generic polymeric materials to form complex nanoparticles for specific usages in the fields of chemistry and biomaterial.^11–18^

DNA, the carrier and transmitter of genetic information in all living systems, consists of four different deoxynucleotide monomers.^5^ Each monomer is formed with a phosphate group, a deoxyribose, and one of four nitrogen-involving nucleobases, while the nucleobases include thymine (T), adenine (A), guanine (G), and cytosine (C).^1,19^ Between the monomers, the phosphate group and the deoxyribose can form the phosphodiester bond, which links a certain number of monomers into a DNA chain, named single-stranded DNA (ssDNA).^1,19^ Based on the principle of Watson–Crick base-pairing, the continuous ssDNA can pair with another one in antiparallel directions to form a double-helix DNA structure, that is double-stranded DNA (dsDNA). Between the two polymeric chains, the nucleobases of C and G can form three hydrogen bonds, and A and T can form two hydrogen bonds, resulting in the base pairs with the certain complementary ssDNA inside the dsDNA.^20–24^

Despite that DNA molecule is composed of simple units, the variety of dsDNA and the programmability of the structure can be realized by accurate design and freely defined constitutions. In artificially synthesized dsDNAs, the quantity and order of the four monomers are accurately arranged as designed, which determines the diversity of dsDNAs. The freely defined constitutions of DNA are based on sequence-specific base attacking interactions, which are determined by both the external conditions and the sequences of DNA duplexes.^25–28^ With the rapid development of nanotechnologies and availability to manage the constructions of DNA nanostructures, a variety of designable principles and plentiful DNA-based rigid titles have been used to prepare various dimensional architectures into rationally assembling nanoparticle templates or biomolecular scaffolds,^29–34^ such as tile assembly,^30,35^ origami structure,^36–39^ and dynamic nanomechanical system.^40–45^ Owing to the inherent nature of biocompatibility and biodegradability of DNA material, different kinds of structural and functional materials based on DNA have been reported with promising potential for many biological usages in different fields.^46–49^ Subsequently, DNA nanotechnology and nanomaterials kept gaining increasing interests in DNA material science and engineering, especially with the development of DNA nanotechnology triggering by the growing demands in biological, chemical, or medical-related usages.^50–56^

DNA nanomaterials were developed with a wide variety of structures, including two- and three-dimension (2D and 3D) constructions. Furthermore, according to the various molecular building-styles of functional DNA materials, the constructions of artificially synthesized DNA nanostructures contain single-layer and multilayer nanostructures, which also can be sorted into linear, circular, and branched form.^57^ The DNA origami technique makes it easy for preparation of DNA, which also promotes the explosive development of structural DNA nanotechnology. The advanced development of DNA nanotechnology then breeds various novel DNA nanomaterials that are applied widely, including tissue engineering, immune engineering, drug delivering, disease diagnosis, and as tools for molecular biology or as biosensors.^54–56,58–67^ However, for therapeutic applications, some multifunctional polymeric DNA nanomaterials still maintain drawbacks, including being easily-metabolized, lack of targeting, and instability.^68–70^ Therefore, the combinations of DNA nanomaterials and other nanoparticles and hyper-polymeric compounds, such as gold nanoparticle (AuNPs),^69,71,72^ polyethylenimine (PEI),^73–77^ chitosan,^78–80^ and poly-l-lysine (PLL),^81–83^ are also developed to overcome those limitations.

While the structural DNA nanotechnology has developed quickly and proved important, the studies on the application of those DNA nanostructures are actually more practical and critical.^29,84–87^ In such efforts, some previous studies demonstrated that some DNA scaffolds with specific structures or other materials could affect cell biological behaviors, such as cell viability,^68,88^ proliferation,^62,63,65,66^ migration,^89^ differentiation,^90,91^ morphology,^92^ and autophagy.^67^ Owing to the special nature, DNA-based materials could be employed in tissue regeneration engineering and potential treatments for some diseases.^81,93–95^ In this aspect, many DNA nanostructures were also combined with other materials, such as nucleic acid aptamer (DNA and RNA),^96–100^ polypeptide,^101–103^ protein,^87,104–106^ and some chemical drugs,^107–110^ in which the DNA nanostructures were designed as delivery systems. When combined with nucleic acid aptamer, the complex DNA-based nanostructures could enhance the ability of targeting,^68,88,100^ inhibition of malignant cells,^100^ and antitumor effects.^88,100,111^ More complex designs of DNA origami enable more advanced applications in other fields, such as in photonics,^112–114^ electronics,^115–117^ reaction networks of artificial enzymes,^118,119^ and etc. This review will summarize the structural design and self-assembly of DNA nanostructures and their biological applications, and discuss the current challenges and future directions.

## Fundamentals of DNA origami design

The structural DNA nanotechnology was pioneered by Ned Seeman and colleagues from 1982.^6^ There arouse an interest in DNA structures, which developed tremendously thereafter.^120–125^ The DNA formed stabilized branch junctions, which can be further modified via sticky ends to form higher-order structures and lattices, is the vital foundation of structural DNA nanotechnology including DNA origami.^6^ As for the intrinsic internal sequence symmetry, the branched DNA structures are naturally mobile,^126^ and the sequence symmetry should be minimized to form stable branched junction.^6,126^ Seeman et al. suggested to possibly use the stale junction to synthesis 3D frameworks of nucleic acids via legitimately designed sticky-end associations. They assembled different multi-arm junctions, including 4-, 5-, 6-, 8-, and 12-arm junctions, demonstrating that DNA junctions could be precisely branched.^127–129^ However, considering the structural flexibility and the unpredictable conformations, multi-arm junctions are not suitable to act as the basic structural elements, which are needed to assemble higher-order periodic lattice structures. To solve this problem, Seeman and colleagues constructed a DNA double-crossover molecule by creating a structural motif with two 4-way junctions, constructing a rigid DNA molecule by two crossovers.^130,131^ In 1990s, a periodic 2D crystalline, the first higher-order DNA lattice nanostructure, was successfully made from crossover molecule.^132^ In 2003, LaBean and colleagues^133^ added crossover molecule into a 4 × 4 DNA title with four different direction arms. The 4 × 4 DNA title has a long central strand to connect four Holliday junctions together into a rigid, branched, and fourfold symmetry, similaring to the crossover molecule with a central strand to link two Holliday junctions by end to end.^133^ The 4 × 4 title reduced the probability of stacking interactions between each arm by placing T_4_ loops at each of the four concerns of the central strand. Nanoribbon or 2D grid-like lattice was successfully synthesized via supporting 4 × 4 title with appropriate sticky end association sites. For the inspiration of the 4 × 4 title and crossover molecule, some DNA basic units, such as three- and six-point star title, and six-helix bundle, were successfully assembled.^134–136^ These DNA basic units can further self-assemble to construct 2D arrays with various patterns.^137^ The 3D macroscopic crystal could also be successful synthesized by the assembly of a rationally designed tensegrity triangle motif.^138^

In addition to synthesizing periodic DNA nanostructures by titles, LaBan et al. also investigated a long scaffold to guide the nucleation of DNA titles into barcode DNA lattices with complicated aperiodic nanostructures.^139^ The long DNA scaffold consisted of shorter synthetic oligos, for adding binding sites to shorter strands and forming larger individual complex with barcode DNA nanostructures, which could be further constructed into latticed nanostructures if different sticky ends provided. Shih et al.^140^ constructed an octahedron using a ssDNA with 1.7 kilobases. Six four-way junctions at each vertex and five double-crossover struts were embedded into the octahedron structure. The synthesis of the octahedron structure includes two steps as follows: (1) five staples with 40 nt bind to the 1699-nt scaffold, assembling a giant branched molecule with bulges at different arms; (2) each arm associating with its partner by the paranemic cohesions interactions, forming the final octahedron-shaped nanostructure.^140^

The fundamental concepts of DNA nanotechnology and previous explorations established a solid foundation, facilitating greatly the development and the design of DNA nanostructures to be more convenient, complex, and diverse.

## Various designs and constructions of DNA nanostructures

### Single-layered DNA nanostructure designs

The DNA origami technology was firstly reported by Paul Rothumend in 2006.^38^ The synthesis of some single-layered, complanate structures, whose structures ranged from triangular, simple rectangular and five-point star shape to intricate smiley faces, each with its unique size, ranges roughly 100 nm in diameter.^38^ The procedures related to the design and self-assembly process have been described in detail by Paul Rothumend et al.^38^ Since then, other studies were inspired and successfully synthesized 2D planar patterns, including a depiction of dolphins and a map of China.^141,142^ Then, the creation of complex curvatures within 2D planar patterns and 3D planar nanostructures were reported by Han et al.^143^ The distances of the consecutive crossovers, which can concatenate adjacent helices, are usually constant along the parallel alignment of DNA helices. Nevertheless, if the distance of the crossovers along the inner helices is less than that of outer helices, the tension could make the outer helices bend. This method was successfully adopted to incorporate DNA into concentric ring or square with rounded angels.^144^

Some previous studies reported the applications of two general strategies to investigate 3D nanostructures, and the 3D nanostructures were based on the single-layered design of double-crossover. The first strategy depends on a second-step folding to build 2D planar DNA nanostructures. However, some 2D planar DNA nanostructures are not completely paired with staples. The 2D planar DNA nanostructure is intentionally left as a single-strand at the certain parts so that some 2D planar DNA nanostructures could form some well-cut pieces, such as squares and triangles. When 2D shapes join in with the matched well-cut pieces, the 2D nanostructures could fold and transform into 3D shapes. For instance, a boxlike DNA nanostructure with the external size of 36 × 36 × 42 nm^3^ had a lid which opened and closed like a box.^145^ A 3D DNA box-shaped structure with a switchable lid was also reported, and the DNA boxlike nanostructure had a unique electron microscopy, which could potentially be used widely including delivering drugs, controlling the function of some single molecules, and molecule computing.^146–148^ Other studies developed a stepwise folding mechanism that was self-assembled by a set of staples, to form a DNA prime and a cuboidal boxlike nanostructure.^148,149^

The second strategy applying to develop 3D DNA nanostructure was used to introduce crossovers and to use plane linkages. The parallel DNA nanostructures did not form the single plane, such as DNA nanotubes.^150^ By using the approach with the in-plane curvature strategy, some tanglesome nanostructures including the 3D sphere, nanosized flask, and football were developed.^151^ The double-crossovers DNA nanostructures are relatively rigid structural motifs. However, when compared to duplex DNA at the large size (such as ~100 nm), the structures of single-layered DNA nanostructure are still flexible.

### Wireframe single-layered designs

Given that the designs were based on the double-crossover motifs, the wireframe single-layered DNA nanostructures that were conducted with different design strategies were formed by aligning DNA helices in the parallel and compact way. If some new motifs are added to the DNA nanostructure, new nanostructures will be achieved, such as multi-arm junctions allowing for the mash style of desired patterns. A square junction was used for creating branched nanostructures, which was connected with the stem of the branch region to only one of the base helices of the desired feature, and allowed to the connecting helix to remain at the right angle.^152^ The first wireframe DNA nanostructure with a gridiron pattern was created by Han et al.^153^ The gridiron pattern consisted of four 4-arm junctions, and they joined in each other to form a square frame.^153^ Connecting some gridiron patterns together with a consecutive strand could result in a series of 2D lattice DNA nanostructures.^153^ Subsequently, Zhang et al.^154^ studied another strategy to make much more intricate wireframe nanostructures with a number of multi-arm junctions. Based on the multi-arm junction, the design algorithm named DAEDALUS was created to fully automate the scaffold routing and staple assignment for some specific target DNA nanostructures.^155^

In addition, Benson et al.^156^ created a new DNA nanostructure with a more open conformation with one helix per edge. The structure is therefore stable under the ionic conditions that were always applied for biological assay. The structure was conducted arbitrary target objects to the triangulated meshes to create DNA nanostructures in a wireframe style by using an alternative way.^156,157^ A single DNA helix rather than two is the majority of the edge designs, which requires the DNA scaffold traveling through all of the edges at once, while a few edges require the DNA scaffold to travel twice. The novel multi-arm DNA structures combined with the DNA origami method for the synthesis of 2D DNA scaffolds with folding into 3D by connection strands on the 2D nanostructures, and the novel multi-arm DNA structures were designed with a scaffold routing whose fundamental structures were the A-trails to form the center of the nanostructures through a computational algorithm.^149,158^ The availability of this method was further demonstrated by means of the successful preparation of a series of meshed 3D nanostructures, such as ball, bottle and stanford bunny. The same design concept was also used to synthesize triangulated DNA origami trusses.^159^

Based on the design concept once adopted, many different approaches can be used to create single-layered DNA nanostructures. In spite of successful formation and high yield, several limitations remain when the DNA structures were used in practical applications. For instance, single-layered DNA nanostructures have structural heterogeneity and conformation, which might restrict the applications in precise addressability.^160^ Owing to the vertex discontinuity of the DNA chains, it is difficult to avoid the structural flexibility in the single-layered wireframe design. In addition, the technologies of single-layered DNA nanostructures also shows the potential of biochemical reactions at the single-molecule level.^161^

### Multilayered designs

Single-layered DNA nanostructures could be aggregated to form multilayered 3D nanostructures with the increase of rigidity, which could overcome some limitations and improve the design methods. In the first study on designing 3D solid DNA nanostructures, the DNA nanostructures were based on a six-helix bundle as the fundamental unit.^162^ Douglas et al.^162^ demonstrated the design and self-assembly process of DNA nanostructures with six different shapes, including monolith, railed bridge, square nut, stacked cross, genie bottle, and slotted cross, and the dimensions with precise controlling ranged from 10 to 100 nm. Douglas et al. also reported the validity of the design method by assembling a monolith, square net, and slotted cross by using a honeycomb alignment. In another study, Ke et al.^163^ reported a new approach to design a multilayer DNA structure with a square lattice, which was based on a four-helix bundle. In this design, the square lattice could be folded into the nanostructures of designed dimensions by one-step annealing process, in spite of the intensive density of DNA helices.^163^ The three-helix bundle was regarded as the basic unit in a triangular lattice.^164^ In this study, Ke et al. reported the successful folding of a multilayer DNA structure with helices arranged on the close-packed hexagonal lattice. This study also presented that hybrid DNA structures could incorporate three different shapes all in one design, including square lattice, honeycomb-lattice, and hexagonal-lattice packing of helices.^164^

More work reported that the multilayered DNA nanostructures could be designed to incorporate more various packaging styles, which possess the programmable curvatures and twists.^165^ In this research, Dietz et al. systematically demonstrated that the strands of DNA with programable self-assembly could form a custom-shaped bundle of tightly cross-linked double helices, and they also created several different shapes of intricate DNA nanostructures via combining multiple curved elements together. Another method combined single-strand DNA flexibility and multilayered nanostructure rigidity to build the tensegrity structures.^166^ Tensegrity, also named tensional integrity, is a structural principle related to the usage of isolated component, indicating a reliable balance between components in pure compression or pure tension for stability.^166^ Liedl et al. successfully synthesized a tensegrity prism that was formed with three multilayered DNA bundle nanostructures as compression components and nine ssDNAs regions as tension cables.^166^

Ke et al.^167^ introduced that the multilayered DNA nanostructures had much better rigidity than that of the single-layered DNA nanostructures. Nevertheless, the longer folding course need to avoid the kinetic traps, and the buffer solution should contain a high concentration of magnesium (Mg^2 + ^), which could suppress the electrostatic repulsion of the negatively charged phosphate backbones between adjacent helices. Furthermore, due to the lower accessibility and shorter nucleation region of folding DNA strands, the yield of the single-layered DNA nanostructures is usually higher than that of the compact multilayered DNA nanostructures. The careful design of the scaffold procedure and staple break positions could boost the folding yield of DNA nanostructures.^167^ The parallel alignments of DNA nanostructures in a layer-by-layer fashion are not necessarily required for the 3D DNA nanostructures. Hong et al.^168^ reported a strategy to design a layered 3D wireframe DNA nanostructure by employing crossover pairs, which could connect neighboring layers of DNA double strands together. The layered crossovers could make the scaffold or helper DNA strands go through different layers and get command of the relative direction of DNA strands within the neighboring layers. In this research, the authors successfully created a nine-layered wireframe DNA nanostructure and reported that the neighboring helices could be controlled within well-defined interlayer angles. The 3D latticed nanostructures could also be constructed by applying 3D point-star junctions. However, it would be a challenge to availably control its relative direction in the interspace of 3D.

The design methodologies of DNA nanostructures depend on the usage of DNA molecules rendering objects. An object can be generated by scientists via using of DNA origami or a 3D modeling software. With the adoptions of DNA origami and 3D modeling software, objects can be folded from the 2D plane, visualized as outlined meshes, or filled with layered lines. The preparation procedures of various DNA nanostructures are conducted under a good framework now: (1) composing an object nanostructure, (2) creating the scaffold style layout and defining the DNA sequence by using a software program,^169,170^ (3) designing the oligoes of the scaffold and staple, (4) enabling DNA self-assembling under a buffer solution with the ramp of temperature, (5) purifying and functionalizing the DNA nanostructure when needed, (6) analyzing and visualizing the DNA nanostructure, and then (7) expanding and exploring the various applications of the DNA nanostructure.^171–173^ Various methods have been created to improve the synthesis processes of DNA nanostructures, such as increasing the production efficiency of DNA nanostructures,^174–178^ packaging the DNA nanostructures in a Mg^2 + ^-free buffer solution with an isothermal environment,^179–181^ and purifying the functionalized DNA nanostructures to wipe off any misfolding nanostructures or polymers.^182–186^ Some other studies have also developed a few methods to apply DNA nanostructures into different experimental conditions, such as the application of photoinduced crosslinking with 8-methoxypsoralen (8-MOP), which could improve the heat resistance of the DNA nanostructures;^187,188^ the application of external signal triggers such as magnetic or electric fields, chemical, light, pH, and temperature, which could control the formation of DNA nanostructures;^189–193^ and the application of imaging and folding DNA nanostructures in hydrated glycholine under isothermal conditions.^194^

## Biological applications of various DNA nanostructures

The final objects of various DNA nanomaterials are the application for different field, such as biomedicine, chemistry, materials, and etc. However, the structural development is way ahead of the applications of DNA nanomaterials. In this part, we will summarize the biological applications of different DNA and DNA-based materials, and discuss the challenges of them. The contents of this part are mainly devided into six small parts shown as follows.

### Tissue engineering

Tissue engineering is a cutting-edge field that study seeded cells, suitable physical and biochemical factors, and biocompatible materials, as well as combinations thereof, to promote the creation of tissue-like structures.^195,196^ Tissue engineering mainly involves cartilage and bone construction, vascular tissue engineering,^197^ nerve tissue engineering, skin tissue engineering,^198,199^ oral tissue engineering, tendon and ligament tissue engineering, corneal tissue engineering, and tissue engineering of some other important organs (e.g., liver, pancreas, kidney, and lung).^200,201^ The therapeutical strategies adopted mainly include seeding cells,^202–205^ synthesizing biological materials,^206–209^ constructing tissues and organs with advanced methods and techniques,^210–213^ and clinical applications.^197,214,215^ At present, there are mainly three ways of tissue repairing commonly used in clinical practice: autologous tissue transplantation, allogeneic tissue transplantation, and artificial substitutes. For tissue regeneration engineering including cell seeding, proliferation, orientation, and differentiation of the stem or progenitor cells on the injured sites, various bio-mimicked materials or medicines with the use of nanotechnology were studied to provide the adhesive surfaces.^216–220^ The nanotechnology can be used to develop and investigate different nanomaterials and medicines, which can provide different substrates for cell seeding, adhesion and growth, to aid the tissue regeneration on the defects.^63,64,221–224^ The most important purpose of regenerative nanomaterials and medicines is to rebuild or create functional tissue replacements to repair the function and morphology of defected tissues.^225^ In this part of the review, we will summarize the applications of DNA nanostructures, discuss the limitations of different studies, and present the gap between the studies and clinical applications.

**Fig. 1:**
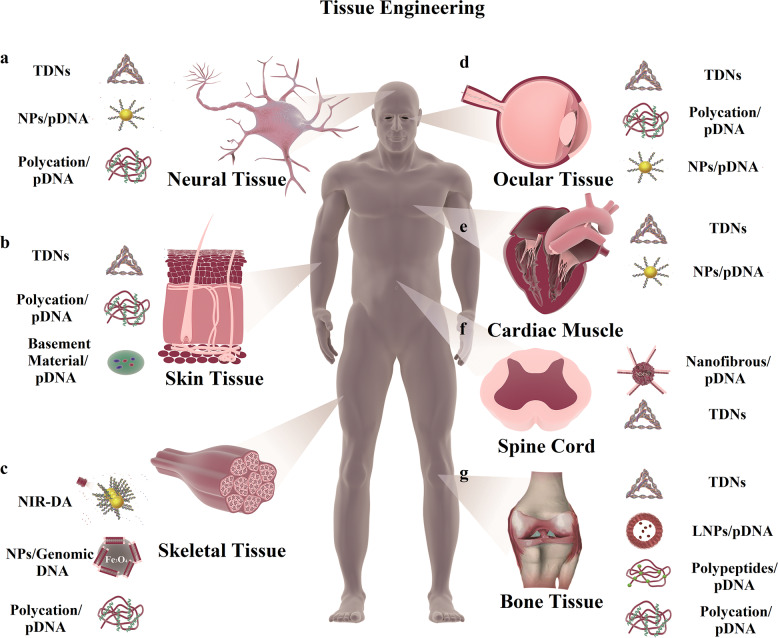
The biological applications of various DNA-based and DNA-encoding nanomaterials in tissue engineering. **a**, **f** The biological applications of different DNA-based nanomaterials in neural tissue (e.g., TDNs, NPs loading various pDNA, polycation loading various pDNA, and some nanofibrous loading pDNA). **b** The biological applications of different DNA-based nanomaterials in skin tissue (e.g., TDNs, polycation loading various pDNA, and some basement materials loading pDNA). **c**, **e** The biological applications of different DNA-based nanomaterials in skeletal and cardiac muscle engineering (e.g., skeletal tissue: NIR-DA, NPs loading genomic DNA, and various polycation loading pDNA; cardiac muscle: nanofibrous loading pDNA and TDNs). **d** The biological applications of different DNA-based nanomaterials in corneal tissue (e.g., TDNs, NPs loading various pDNA, and polycation loading various pDNA). **g** The biological applications of different DNA-based nanomaterials in bone tissue engineering (e.g., TDNs, LNPs loading pDNA, polypeptides loading various pDNA, and polycation loading various pDNA)

With the rapid development of DNA nanotechnology, the applications of DNA nanostructures in tissue regeneration engineering are embodied in altering biological behaviors of cells, such as morphology, viability, growth, and migration.^226,227^ The potential applications of DNA nanostructures in tissue regeneration include the regeneration of bone and cartilage (Fig. 1g),^65,228,229^ nervous system (Fig. 1a, f),^63,64,230^ and integumentary system (Fig. 1b),^231–236^ as well as changing the stress response of tissue, such as anti-inflammatory effects,^237,238^ anti-aging effects,^239^ and anti-apoptosis (Fig. 1).^240^ Owing to the interesting feature of DNA with the ability to intercalate synthetic or natural molecules, such as daunorubicin, amsacrine, and adriamycin, the combinations of DNA nanostructures and other molecules are regarded as complex drug delivery systems (DDSs) for many active and photosensitive compounds (Table 1).^241–245^ Therefore, the DNA-based nanostructures or nanomedicines are exerting their effects on tissue regeneration engineering.^246–248^

**Table 1:** The biological applications of various DNA-based and DNA-encoding nanomaterials in tissue engineering

Tissue regeneration engineering	Material format	Composition	DNA nanotructure	Biological function	References
Bone tissue regeneration	DNA/chitosan nanoparticles	1. Poly(d, l-lactic-co-glycolic acid)/hydroxylapatite (PLGA/HAp) 2. pDNA/*BMP-2*	pDNA/*BMP-2*	PLGA/HAp composite scaffolds could deliver the pDNA/*BMP-2* into human marrow stem cells (hMSCs), which showed the higher cell attachment, higher cell viablity and desirable transfection efficiency of DNA.	^275–279^
	*pBMP-2*/Polyethylenimine nanoparticles	1. PEI 2. Human pDNA/*BMP-2*	Human pDNA/*BMP-2*	Bone formation.	^282,283^
	DNA-loaded calcium phosphate nanoparticles	1. Nano-calcium phosphate paste 2. pDNA/*BMP-7* 3. pDNA/*VEGF-A*	1. pDNA/*BMP-7* 2 pDNA/*VEGF-A*	Enhanced regeneration of bone volume and a significantly faster healing.	^255,284^
	Chitosan-gold nanoparticles mediated gene delivery of *c-myb*	1. Chitosan-gold nanoparticles 2. pDNA/*c-myb*	pDNA/*c-myb*	Suppressing osteoclastogenesis and promoting osteogenesis of dental implant even in osteoporotic condition	^256,285^
	CS/CSn(pDNA/*BMP2*)-GP	1. chitosan (CS)-based hydrogel with a,b-glycerophosphate (a,b-GP) 2. pDNA/*BMP-2*	pDNA/*BMP-2*	Enhancing cell proliferation, maintaining great potential in gene delivery system and tissue regeneration of periodontium	^286–290^
	Chitosan/β-glycerophosphate (CS/β-GP) hydrogel as a *VEGF*-sustained release system	1. Chitosan/β-glycerophosphate (CS/β-GP) hydrogel 2. *VEGF* gene	*VEGF* gene	Delivering and releasing *VEGF*, and promoting cell proliferation and differentiation of dental pulp stem cells (DPSCs).	^291^
	Lipopolysaccharide-amine nanopolymersomes (LNPs) modified with *Noggin* small interfering (si)RNA (*siNoggin*) and pDNA/*BMP-2*	1. Lipopolysaccharide-amine nanopolymersomes 2. *siNoggin* 3. pDNA/*BMP-2*	1. *siNoggin* 2. pDNA/*BMP-2*	Enhancing osteogenic differentiation.	^257^
	Star-shaped poly(l-lysine) polypeptides/genomic DNA	1. Star-shaped poly(l-lysine) polypeptides 2. pDNA/*VEGF* and pDNA/*BMP-2*	1. pDNA/*VEGF* 2. pDNA/*BMP-2*	Irritating mesenchymal stem cells (MSCs) to differentiate into bone tissue.	^292^
	DNA/protamine complex scaffold	1. Protamine 2. pDNA/*FGF-2* or pDNA/*BMP-2*	1. pDNA/*FGF-2* 2. pDNA/*BMP-2*	Enhancement of bone regeneration, and gene delivery.	^293,295^
	Tetrahedral DNA nanostructures (TDNs)	TDNs	TDNs	Enhancing the cell growth and motility of different cells, such as adipose stem cells (ASCs), human periodontal ligament stem cells (PDLSCs), MSCs, and chondrocytes. TDNs could act the anti-inflammatory effects on the periodontist model, and could promote osteogenic differentiation	^65,226,296–299,301^
	TDNs/Wogonin	1. TDNs 2. Wogonin	TDNs	Enhancing expression of chondrogenic markers, downregulating matrix metalloproteinases and inflammatory mediators, and promoting the expression of tissue inhibitor of metalloproteinase 1 and B-cell lymphoma 2.	^302^
Neural tissue engineering	Nanofibrous modified with pDNA	1. A nanofibrous micro cell carrier 2. A self-assembled nano-sized polymer 3. pDNA/*NR4A1*	pDNA/*NR4A1*	Promoting intervertebral disc (IVD) regeneration and suppress the fibrosis.	^94^
	A novel DNA-based chemical approach with the synthetic DNA-binding inhibitor	Pyrrole-imidazole polyamides (PIPs)	N/A	Targeting cell differentiation controlling genes of human induced pluripotent stem cells (hiPSCs), and the DNA-binding inhibitor could target the *SOX2* gene.	^341^
	DNA aptamer	yly12 aptamer	yly12 aptamer	The aptamer yly12 could bind neural cell adhesion molecule L1 (L1CAM), which was expressed in normal neural tissue as surface antigen.	^342^
	Poly(lactide-co-glycolide) (PLG) delivery DNA complexes	1. PLG 2. pDNA	pDNA	Promoting the transgene expression and the regeneration of complicated neural system.	^332^
	Nanoparticles loading pDNA and RNA	1. Nanoparticles 2. pDNA or RNA (e.g., siRNA)	1. pDNA 2. RNA (e.g., siRNA)	Delivering genetic materials into neural stem cells (NSCs) and guiding cell differentiation.	^343–347^
	TDNs	TDNs	TDNs	1. Enhancing cell proliferation, migration, and neuronal differentiation of NSCs. 2. Improving the recovery of motor function and the neural tissue regeneration in the injured site of spine cord. 3. Potential neuroprotective effects on the cell models of Alzheimer’s Disease (AD) and Parkinson’s Disease (PD).	^63,64,229,239,329,348^
Skeletal and cardiac muscle engineering	Near-infrared light-activated DNA agonist (NIR-DA) nanodevice	1. Nongenetic manipulation 2. DNA agonist	DNA agonist	Delivering the cell signaling and regulating the receptor tyrosine kinase (RTK) signaling, cell migration, and myogenesis of skeletal muscle satellite cells.	^89^
	Iron-oxide nanoparticles loading genomic DNA	1. Magnetic nanoparticle 2. Genomic DNA	Genomic DNA	Capturing some chemotherapy agents from human serum, including cisplatin, epirubicin, and doxorubicin (DOX), and protecting cultured cardiac myoblasts from the lethal levels of chemotherapy agents	^363^
	TDNs	TDNs	TDNs	Cardio-protection effects from myocardial ischemia-reperfusion injury (MIRI) by taking the advantage of the anti-inflammatory and antioxidative potential.	^365^
Skin tissue engineering	N,N,N-trimethyl chitosan chloride (TMC)/pDNA-*VEGF* complexes	1. TMC 2. pDNA-*VEGF*	pDNA-*VEGF*	Promoting the expression of *VEGF* gene and wound healing.	^388^
	Collagen-chitosan scaffold/silicone membrane bilayer dermal equivalent (BDE)/ pDNA-*VEGF*	1. BDE 2. pDNA-*VEGF*	pDNA-*VEGF*	Effective transfection abilities to promote the expression of *VEGF* gene.	^390,391^
	Hydrogel embedded with resveratrol (Res) and pDNA-*VEGF* (Gel-Res/pDNA-*VEGF*)	1. Gel-Res 2. pDNA-*VEGF*	pDNA-*VEGF*	Promoting the healing of splinted excisional burn wounds.	^392^
	Nanofibers loading pDNA-*bFGF* and pDNA of angiopoietin (pDNA-*ANG*)	1. PLLA/POSS 2. pDNA-*bFGF* 3. pDNA-*ANG*	1. pDNA-*bFGF* 2. pDNA-*ANG*	Promoting revascularization of the deep skin defect healing.	^231^
	Electrospun core-sheath fibers loaded with pDNA-*bFGF*	1. Electrospun core-sheath fibers 2. pDNA-*bFGF*	pDNA-*bFGF*	Promoting skin regeneration in diabetic rats.	^393^
	TDNs	TDNs	TDNs	1. TDNs (sizes ≤ 75 nm) could effectively penetrate the skin of mice and human, and reach dermis layer, which could load and deliver DOX to subcutaneous tumor site. 2. Promoting fibroblast (HSF cell line) and keratinocyte (HaCaT cell line) growth and migrating.	^230,398^
Other tissue engineering	N, N, N-trimethyl chitosan chloride (TMC) modified with pDNA-*VEGF*	1. TMC 2. pDNA-*VEGF*	pDNA-*VEGF*	Enhancing the angiogenesis.	^400^
	Scaffolds containing both pDNA-*VEGF* and pDNA-*FGF2*	1. Scaffolds (e.g., collagen-heparin scaffolds) 2. pDNA-*VEGF* and pDNA-*FGF2*	pDNA-*VEGF* and pDNA-*FGF2*	Enhancing the angiogenesis.	^401,402^
	Aptamer-conjugated hydroxyapatite (Apt-HA)	Apt-HA	Apt-HA	Promoting angiogenesis and bone regeneration.	^399^
	TDNs	TDNs	TDNs	1. Promoting angiogenesis in vitro and the model of the jaw bisphosphonate-related osteonecrosis. 2. Facilitating the corneal wound healing, promoting the re-epithelialization of wounds, and improving the corneal transparency. 3. Preventing retina ischemia-reperfusion injury from the oxidative. 4. TDNs could induce the immune tolerance and prevent the onset of Type 1 diabetes.	^62,403–406,411^
	PEI-DNA nanoparticles	1. PEI 2. Genomic DNA	Genomic DNA	Delivering corneal gene for the corneal gene therapy.	^408^
	DNA-based carrier systems	DNA nanoparticles	DNA nanoparticles	Ophthalmic drug delivery.	^407^
	AuNPs-encoding pDNA	1. AuNPs 2. pDNA	pDNA	Transfecting human retinal pigment epithelium cells.	^409^

#### Bone tissue engineering

The loss and dysfunction of bone tissue, which are mainly caused by aging, injury, inflammation, such as rheumatoid arthritis (RA) or osteoarthritis (OA), and tumors, are associated with individual susceptibility, and can result in severe morbidity and socio-economic issues.^249,250^ In the skeletal tissue, the DNA nanostructures are mainly used to provide the adhesive scaffolds or delivery some biological agents to the injured articulations, and the DNA nanostructures as the delivery system could overcome some drawbacks of other biological agents, such as poor penetration level and low stability.

At the first decades, the main application of DNA nanostructures in bone tissue was gene therapy by using plasmid DNA (pDNA).^215,226,251–253^ In previous studies, the pDNA or other DNA nanomaterials were usually designed as the delivery systems to transport some growth factors and genes, including fibroblast growth factor (FGF), bone morphogenetic protein (BMP), and vascular endothelial growth factor (VEGF),^254–258^ which was regarded as viral gene delivery (Fig. 1g).^259^ The combinations with different materials and seed cells were also conducted in a vast of studies, and the purpose was to promote the seed cell proliferation and differentiation into the functional target tissues including bone and cartilage.^260–264^ The treatments based on different recombinant human growth factors have been investigated and the therapeutic effects were encouraging. Owing to the short half-lives of recombinant human growth factors and the needed for clinical effectiveness, supraphysiological dosages were used, and their production was expensive.^265,266^ The supraphysiological dosages of recombinant human growth factors could also cause several side effects, such as ectopic formation of bone tissue and soft tissue swelling.^267^ Therefore, the non-viral gene therapy then became a promising alternative strategy for bone tissue repair.^268–270^

In studies within recent decade, the non-viral gene vectors were developed by combining DNA with other scaffolds.^259,271–275^ The complex of DNA and poly(lactic-co-glycolic acid) (PLGA)/HAp composite scaffolds was constructed to delivery pDNA-*BMP-2* into bone marrow stem cells (BMSCs) and enhance the DNA transfection efficiency, which was quite encouraging and promising for bone regeneration.^276–280^ Previous studies reported that the delivery of *FGF-2* and *BMP-2* genes could upregulate the transcription of *Runx2* and *osteocalcin,*^281,282^ and Salem et al. reported that non-viral gene delivery of *FGF-2* and *BMP-2* via pDNA could attenuate the harmful effects of diabetes mellitus on bone regeneration at the defect sites.^254^ PLGA was used to encapsulate the novel nanoparticles that consisted of polyethylenimine (PEI) and human pDNA-*BMP-2* to create p*BMP-2*/PEI nanoparticles for bone formation.^283,284^ The *BMP-7* and *VEGF-A* genes were encoded into the injectable DNA-loaded nano-calcium phosphate paste, and the nanocomplex was regarded as bioactive bone substitution material.^256^ The DNA-loaded bone paste encoding with *BMP-7* and *VEGF-A* showed enhanced regeneration of bone volume and significantly faster healing in critical site of bone loss at the early stages.^256^ The DNA-loaded nanoparticles could release DNA, resulting in cell transfection, and then promote the expression of BMP and VEGF in bone cells.^285^ Takanche et al. found that pDNA-*c-myb* conjugated with chitosan-gold nanoparticles (Ch-GNPs/*c-myb*) could suppress osteoclastogenesis and promote osteogenesis of dental implant even in osteoporotic condition, making an applicable material to sustain dental implant integration and therapy in some degenerative bone diseases.^257,286^ Some studies loaded *BMP-2* gens (pDNA-*BMP-2*) into a chitosan (CS)-based hydrogel with a,b-glycerophosphate (a,b-GP), termed CS/CSn(pDNA-*BMP2*)-GP. The CS/CSn(pDNA-*BMP2*)-GP then possessed excellent capability in enhancing cell proliferation, maintaining great potential in gene delivery system and tissue regeneration of periodontium.^287–291^ Chitosan/β-glycerophosphate (CS/β-GP) hydrogel as a *VEGF*-sustained release system could delivery and release *VEGF*, and promote cell proliferation and differentiation of dental pulp stem cells (DPSCs).^292^ Huang et al.^258^ applied lipopolysaccharide-amine nanopolymersomes (LNPs) to delivery *Noggin* small interfering (si)RNA (*siNoggin*) and pDNA-*BMP-2* to transfect cells, respectively. This study demonstrated that LNPs/*siNoggin* and LNPs/*pBMP-2* could both enhance the osteogenic differentiation, and the osteogenic differentiation of LNPs/*siNoggin* was better than that in LNPs/*pBMP-2.* Moreover, different structural variations of star-shaped poly(l-lysine) polypeptides were used as promising nano-viral gene delivery systems for mesenchymal stem cells (MSCs), which could irritate MSCs to differentiate into bone tissue by delivering pDNA-*VEGF* and pDNA-*BMP-2.*^293^ Novel DNA/protamine complex scaffold was developed for a number of clinical usages, such as enhancement of bone regeneration,^294,295^ and pasting and delivery the genes of pDNA-*FGF-2* or pDNA-*BMP-2.*^294,296^

Beyond the application of pDNA in bone tissue regeneration, some other DNA nanostructures were also developed for bone regeneration with their own features in recent decades. Some functional DNA materials were synthesized to promote the reconstruction of bone tissue, and some DNA materials could deliver drug monomers to exert anti-osteoarthritis effects. Tetrahedral DNA nanostructures (TDNs) have the specific framework and can be internalized by different cells, in which they can change the cell biological behaviors (Fig. 1g). Lin et al. reported that TDNs could enhance the cell growth and motility of different cells, such as adipose stem cells (ASCs), human periodontal ligament stem cells (PDLSCs), MSCs, and chondrocytes.^65,227,297–300^ TDNs could also induce stem cells toward osteogenic differentiation.^65,297^ Zhou et al.^301^ also reported that the TDNs could act the anti-inflammatory effects on the periodeontitis model, and could promote osteogenic differentiation. In this study, the TDNs acted the effects of anti-inflammations and regeneration on bone tissue, and the results suggested that DNA nanomaterials might be applied for some inflammatory diseases. In a recent study, TDNs were employed as the delivery loaded with Wogonin to form the TDNs/Wogonin complexes.^302^ The TDNs/Wogonin complexes were verified as a potential nanomedicine for osteoarthritis by executing different biological effects, including enhancing expression of chondrogenic markers, downregulating matrix metalloproteinases and inflammatory mediators, and promoting the expression of tissue inhibitor of metalloproteinase 1 and B-cell lymphoma 2. The TDNs/Wogonin thus proved itself with great potential as an injectable nanomedicine in the treatment of osteoarthritis.

**Fig. 2:**
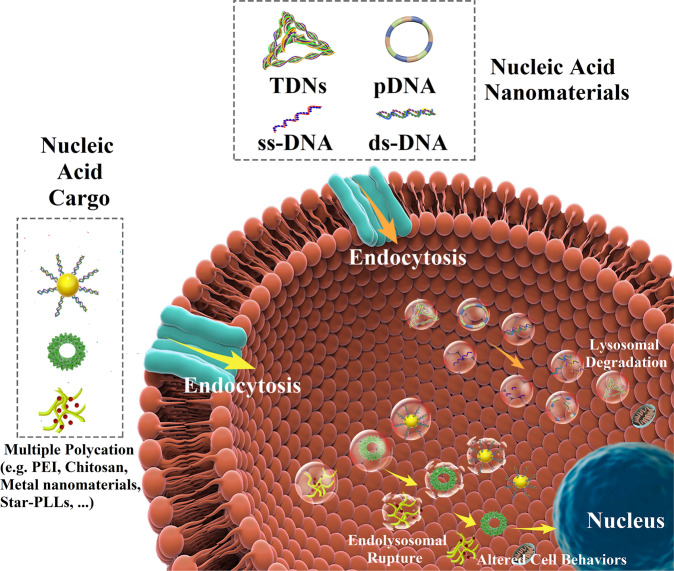
The fate of DNA materials following delivery into cells. Nucleic acid nanomaterials (e.g., TDNs, pDNA, ssDNA, and dsDNA) with the excellent ability to enter cells, can be internalized by cells and degraded by lysosomal. Nucleic acid cargos combine the DNA materials and different delivery systems. These vectors can protect the DNA materials from degradation and promote cellular internalization. When inside the cells, these vector-DNA material complexes are embedded in an endosome, and these vectors can help DNA materials to escape lysosomal degradation

The DNA materials applied for bone regeneration were mainly the combinations of pDNA and other materials for enhancing the gene transfection efficiency to turn gene on or off, and the pDNA used was usually adenovirus plasmid.^303,304^ In general, for the easy laboratory manipulation, considering the features of porosity, adjustable degradation rate, and good mechanical property of natural and synthetic polymers, vast polymers were used to modify DNA materials for bone regeneration, such as alginate, chitosan, proteins (eg. fibrin, silk, and collagen), glycosaminoglycans, polylactides (PLA), polyglycolides (PGA), co-polymers PLGA, polycaprolactone (PCL), and polyethylene-glycol (PEG).^305–308^ When these complex materials were used in cells or organs, cellular uptake via endocytosis is the first challenge, since the efficiency and process of cellular uptake depend on the cell types and the physicochemical properties of the complex materials, such as the surfaces of cell and materials, and the shape and size of materials.^309,310^ The mechanisms of endocytosis are diverse, including both caveolae and clathrin dependent, micropinocytosis, and independent mechanisms.^311^ For DNA nanomaterials without any modification, when they are internalized by cells, the first existing form within cells is early endocytic vesicles, which can be rapidly acidified and thereby form late endosomes, and then DNA nanomaterials will be degraded by lysosomes (Fig. 2).^312^ Those nano-viral vectors mentioned above have some excellent properties, such as being non-immunogenic, high cellular uptake, outstanding endosomal escape, and rapid gene release.^313–316^ However, these studies mainly focused on the gene delivery and release, the investigations of the biological application in bone regeneration were limited in cytology experiments and small animal experiments, primarily the pre-clinical trials. The gene release processes of some delivery systems were even unclearly conducted and demonstrated. More importantly, the underlying mechanisms and the biosafety of these DNA materials combined with other scaffolds in bone regeneration remain poorly understood, and the effects on bone tissue regeneration of these nanomaterial complexes should be elucidated in further investigations.

#### Neural tissue engineering

Neural tissue is considered as the vital system for all lives.^317^ For human being, neurodegenerative disorders and damages to neural tissue can result in severe and permanent disabilities,^317,318^ bringing heavy burdens for individuals, families, and socity.^319^ Owing to the lack of regeneration ability and complicated connections between different physiology systems, it is a great challenge to recuperate injured neural system, and make it work normally.^317,320–322^ Therefore, neural tissue engineering has emerged as a potential remedy with great capacity in repairing and regenerating nerve tissue injuries.^323^ In recent decades, biocompatible materials have been largely employed to achieve the regeneration and functional recover of injured neural tissue. Some successfully synthesized nanomaterials can provide mechanical support for neurite growing and inhibit the formation of scar tissue, affect biological cues to steer the growth of axons, promote regeneration, and incite the integration with the resident healthy tissue.^321,324^ Currently, the natural (e.g., collagen, gelatin, hyaluronic acid, and alginate) and synthetic nanomaterials (e.g., PLA, PGA, PLGA, and PEG) are considered as neural scaffolds for their biocompatible and biodegradable properties.^325–329^ Those nanomaterials could also combine with some specific DNA materials to obtain other positive effects for gene delivery or cell activation in neural tissue regeneration (Fig. 1a, f).^240,330–333^

The applications of gene therapy with DNA materials in neural tissue regeneration are also significant, and the pDNA is the most common material used.^331,332,334^ Feng et al.^94^ reported an injectable two-stage gene delivery system, which consisted of a nanofibrous micro cell carrier and a self-assembled nano-sized polymer, and found that the combination of the two-stage pDNA delivery system could promote intervertebral disc (IVD) regeneration and suppress the fibrosis (Fig. 1f). In this study, the pDNA was loaded with orphan nuclear receptor 4A1 (*NR4A1*). The *NR4A1* was demonstrated as a safe and efficient gene agent with pleiotropic regulatory function to restrain pathogenic fibrosis, which was associated with vascular homeostasis,^335,336^ inflammatory responses,^337,338^ and metabolism of lipid and glucose.^339–341^ Taniguchi et al.^342^ reported a synthetic DNA-binding inhibitor with the ability of targeting vital cell differentiation controlling genes, and demonstrated that the DNA-binding inhibitor could target the *SOX2* gene. The downregulation of *SOX2* gene could trigger pluripotent stem cells differentiate into the mesoderm, which could form the notochords to wrap neural tubes. Wang et al.^343^ developed a DNA aptamer, named yly12, which could strongly target neurites. The aptamer yly12 could bind neural cell adhesion molecule L1 (L1CAM), which was expressed in normal neural tissue as surface antigen. Therefore, the aptamer yly12 could be used as a potential molecular probe to investigate the development of nervous system and the diagnosis of neurological diseases. Shea et al. used the poly(lactide-co-glycolide) (PLG) delivery DNA complexes to achieve gene delivery.^333^ In this study, the neurons and accessory cells were co-cultured together to measure the outgrowth progress of neurites when the PLG-DNA substrates were applied. Shea et al. found that surface immobilization of PLG-DNA substrates, which were modified with pDNA as the gene carrier and deliver systems, could promote the transgene expression and the regeneration of complicated neural system. In other studies, some nanoparticles (e.g., magnetic nanoparticles (MNPs), magnetic core-shell nanoparticles (MCNPs), and gold nanoparticles (AuNPs)) loading pDNA or RNA (e.g., short interfering RNA (siRNA)) were used to deliver genetic materials into neural stem cells (NSCs) and guide cell differentiation.^344–348^ Till now, although the above-mentioned work has presented numerous nanomaterials modified with different DNA materials, their applications in neural tissue engineering are still greatly limited, becasue the work is at the initial stage and the results are prelimilary. The mechanisms of different DNA-based and DNA-modified nanomaterials studied in neural tissue regeneration remains unclear. However, these studies intiated the step by providing the design principles for DNA-based and DNA-modified nanomaterials that were used in in vitro and in vivo nerve regeneration.

Recently, TDNs were reported as promising DNA nanomaterials for neural tissue regeneration (Fig. 1f).^63,64,230^ In these studies, it was demonstrated that NSCs treated with TDNs could exhibit enhanced cell proliferation, migration, and neuronal differentiation.^63,64^ In the in vivo experiment, a concomitant approach of NSCs and TDNs was applied in the animal model of spine cord injury (SCI).^230^ The study found that the TDNs possessed the capacity to increase the cell survival of the transplanted NSCs, and the concomitant approach of NSCs and TDNs could improve the recovery of motor function and the neural tissue regeneration in the injured site of spine cord. These results provided an interesting treatment strategy for neural tissue regeneration. Furthermore, Lin et al. also reported potential neuroprotective effects of TDNs on the cell models of Alzheimer’s Disease (AD) and Parkinson’s Disease (PD).^240,330,349^ Although these results were prelimilary, they might provide a promising synergistic agent for treating nerve damage and degenerative diseases. For futher studies, we would believe that TDNs and other DNA nanomaterials might be used to deliver some functional drugs and DNA/RNA sequences to the neural tissue for disease therapy.

#### Skeletal and cardiac muscle engineering

The obligations of skeletal and cardiac muscle are to support the body movements and blood circulation, where the chemical energy of nutrients are turned into kinetic and heat energy.^350^ Although the skeletal and cardiac muscle are both differentiated from the mesoderm, physiology and morphology properties of them are dissimilar.^351^ Lack of regeneration ability, the adult mammalian cardiac muscle could not self-renew when some injuries happen, such as heart attack.^352–354^ In contrast, skeletal muscle tissue can naturally repair minor injuries by self-regenerating, thanks to the property instigated by the activation of residual stem cells named satellite cells.^355–357^ However, severe damage and some myopathies can result in irreversible loss of muscle and functional disorder.^206,358,359^ Current therapies of broken skeletal muscle and some myopathies include injection of in vitro cultivated muscle cells and autologous skeletal muscle transplantation, but with many limitations in clinical applications of treating patients.^206,360–362^ In some previous studies, it was reported that some biomaterials could stimulate skeletal muscle regeneration via supplying chemical and physical cues to muscle cells, which could mimic the natural cascade of muscle tissue regeneration.^206,212,359,363^ The possibility of skeletal muscle regeneration is of great promise in curing the movement disability of bones and muscles,^89^ and novel medicines/biomaterials with some positive effects on cardiac muscle, such as protective effects from drug toxicity and hypoxia injury, are also of great significance in treating heart diseases.^364^

In recent studies, DNA-based nanomaterials were used for regenerating skeletal muscle and exerting protective effects from hypoxia injury of cardiac muscle, respectively.^89,365^ Wang et al.^89^ creatively developed a fancy near-infrared light-activated DNA agonist (NIR-DA) nanodevice (Fig. 1c). The NIR-DA system could deliver the cell signaling by nongenetic manipulation, and change the tissue phenotype of deep sites by activating the receptor tyrosine kinase (RTK), which enables the control of cell polarization, cytoskeletal remodeling, and directional migration. The study demonstrated that NIR-DA system could be employed in vivo to regulate RTK signaling, cell migration, and myogenesis of skeletal muscle satellite cells. Therefore, the NIR-DA system was regarded as a promising regeneration medicine for skeletal muscle injury.

Blumenfeld et al.^364^ reported a new-style covalent attachment of genomic DNA to iron-oxide nanoparticles. These magnetic nanoparticle complexes could capture some chemotherapy agents from human serum, including cisplatin, epirubicin, and doxorubicin (DOX). It was found that the DNA-coated nanoparticle complexes could protect cultured cardiac myoblasts from the lethal levels of chemotherapy agents, which could be developed as substrates for medicine capturing. Zhang et al. found the cardio-protection effects from myocardial ischemia-reperfusion injury (MIRI) by taking the advantage of the anti-inflammatory and antioxidative potential of TDNs (Fig. 1e), which could suppress the expression of reactive oxygen species (ROS) and then weaken the oxidative damage and apoptosis of cardiomyocytes.^366^ These studies offered some new ideas of using DNA-modified nanomaterials for treating cardiovascular and cerebrovascular diseases.^367,368^ Although the applications of DNA-based nanomaterials are few in this aspect, an innovative research design idea was provided by these studies for the skeletal and cardiac muscle engineering.

#### Skin tissue engineering

Skin, the biggest organ covering the entire body, consists of the epidermis and the dermis, with a complex blood supply and nerve innervation.^369,370^ For the resident stem cells in skin, the functions include hair regeneration, epidermal homeostasis, barrier resistance, and wound repair.^371,372^ Therefore, the health of skin organ is a significant element for human.^373–375^ The injuries of skin commonly happen with causes including burn, surgical operation, traumatism, chronic skin disease (e.g., parapsoriasis guttata and vitiligo), aging, systemic diseases (e.g., chronic kidney disease (CKD), hypertension, diabetes, and rheumatologic or inflammatory disease), infection (bacterial or fungal) and dermatitis.^376–381^ Nowadays, promoting the wound healing in skin is hot in clinical research, which can be affected by the interaction of several cells, growth factors, and cytokines.^382–384^ Some biomaterials have the abilities of the biomedical functions and tissue regeneration engineering, including implantable devices and some drug and gene delivery.^385–387^ Furthermore, some biomaterials were combined with DNA nanomaterials to overcome some shortcomings and enhance positive effects of these materials in biological application.^388,389^ DNA biomaterials applied for skin regeneration have also been widely studied in different skin diseases (Fig. 1b).^232,388–390^

For the therapy of full-thickness burn wounds, Gao et al.^389^ used the pDNA encoded *VEGF*-165/N,N,N-trimethyl chitosan chloride (TMC) complexes to load a bilayer porous collagen-chitosan/silicone membrane dermal equivalents (BDEs). The cells treated with TMC/pDNA-*VEGF* complexes could highly express *VEGF* gene and quickly promote wound healing. For diabetic chronic wounds, Lou et al.^391^ put the gene of pDNA-*VEGF* into a collagen-chitosan scaffold/silicone membrane bilayer dermal equivalent (BDE), and the DNA complexes showed the effective transfection abilities to promote the expression of *VEGF* gene, which could then promote the angiogenesis and change the immunomodulation.^392^ This study showed that the gene-activated bilayer dermal equivalents (Ga-BDEs) possessed the versatile potentials for accelerating wound healing of diabetic chronic wounds.^391^ Furthermore, Li et al. used hydrogel embedded with resveratrol (Res) and pDNA-*VEGF* (Gel-Res/pDNA-*VEGF*) to promote the healing of splinted excisional burn wounds. The Gel-Res/pDNA-*VEGF* was proved to possess the abilities to enhance the formation of microvascular and suppress the inflammation response.^393^ Additionally, the pDNA of basic fibroblast growth factor (*bFGF*; pDNA-*bFGF*) was also widely studied.^232,390,394–396^ Li et al.^232^ applied nanofibers (poly(l-lactic acid)/polyhedral oligomeric silsesquioxane (PLLA/POSS)) to load pDNA-*bFGF* and pDNA of angiopoietin (pDNA-*ANG*). The PLLA/POSS/*pANG*/*pbFGF* (Fab) composite nanofibers presented the capacities to promote revascularization of the deep skin defect healing. As reported, the electrospun core-sheath fibers loaded with pDNA-*bFGF* could promote skin regeneration in diabetic rats.^394^ Furthermore, dual delivery systems of the pDNA-*VEGF*, pDNA-*bFGF*, or pDNA-*ANG* were applied to promote regeneration of different skin defects.^395–398^

Fan et al. reported that TDNs (sizes ≤ 75 nm) could effectively penetrate the skin of mice and human, and reach dermis layer, which could load and deliver DOX to subcutaneous tumor site.^399^ This study inspired that TDNs could deliver other drugs to treat more subcutaneous tumors. Zhu et al.^231^ demonstrated that TDNs could promote fibroblast (HSF cell line) and keratinocyte (HaCaT cell line) growth and migrating. In addition, TDNs could promote the secretion of VEGF and bFGF and enhance the wound healing. Therefore, TDNs might be an novel DNA materials with the potential in skin tissue regeneration and drug delivery for subcutaneous tumors.

#### Other tissue engineering

Beyond above-mentioned tissue engineering applications, the DNA materials also have been used in vascular tissue and ocular tissue.^62,400–404^ Angiogenesis is important for other tissue regeneration, such as bone, muscle, and neural tissue. Abundant blood supply can enhance the nutrition supply for different tissue regeneration. For angiogenesis, materials loading pDNA or DNA aptamer are widely studied. Mao et al.^401^ used N,N,N-trimethyl chitosan chloride (TMC) that was modified with pDNA-*VEGF* to promote *VEGF* gene expression and thereby effectively enhance the angiogenesis. The increased angiogenesis could also be promoted by some scaffolds containing both pDNA-*VEGF* and pDNA-*FGF2.*^402,403^ Son et al.^400^ developed aptamer-conjugated hydroxyapatite (Apt-HA) to promote angiogenesis and bone regeneration. In recent years, TDNs were employed to promote angiogenesis in vitro and the model of the jaw bisphosphonate-related osteonecrosis.^405–407^ Although the DNA-based complexes applied for angiogenesis are in their early stages, DNA-based materials might be developed as regeneration medicine to promote angiogenesis in different tissues.

As for the research on ocular tissue, the number of studies keeps increasing in recent years, indicating growing interests and great potential of applications for ocular tissue regeneration and ophthalmic drug delivery (Fig. 1d). Heremann et al. combined DNA and nanoparticles as DNA-based complexes to treat eye infections, which was the first report describing the usage of DNA-based carrier systems in ophthalmic drug delivery.^408^ Then, PEI-DNA nanoparticles were employed to deliver corneal gene, with great efficiency in gene delivery for the corneal gene therapy.^409^ AuNPs-encoding pDNA were used to transfect human retinal pigment epithelium cells.^410^ Furthermore, TDNs were used to facilitate the corneal wound healing, promote the re-epithelialization of wounds, and improve the corneal transparency.^62^ Cai et al. reported that TDNs could prevent retina ischemia-reperfusion injury from the oxidative by reducing the ROS.^404^ Moreover, Gao et al.^411^ proven that TDNs could induce the immine tolerance and prevent the onset of Type 1 diabetes (T1D). For T1D occurring with T cell-mediated autommune, inhibition of autoreactive T cell and inducing regulatory Tregs to rebuild the immune tolerance are the two promising strategies. In this study, the researchers reported that the TDNs could control the level of glycemis, protect β-cell, and prevent the onsed of T1D by regulating the immunity. Therefore, some DNA nanomaterials might possess the abilities of immunomodulation for some immune diseases. The regeneration and protection of the ocular tissue are vital for human healthcare, and DNA-based nanomaterials will maintain attractive in this field in the following years.

#### Drug delivery and tumor therapy

In recent decades, DNA nanotechnology has also made amazing progress in biomedical applications in drug delivery and tumor therapy. In this part of the review, the development trajectories and latest advances of pristine DNA-based nanostructures will be highlighted.

**Fig. 3:**
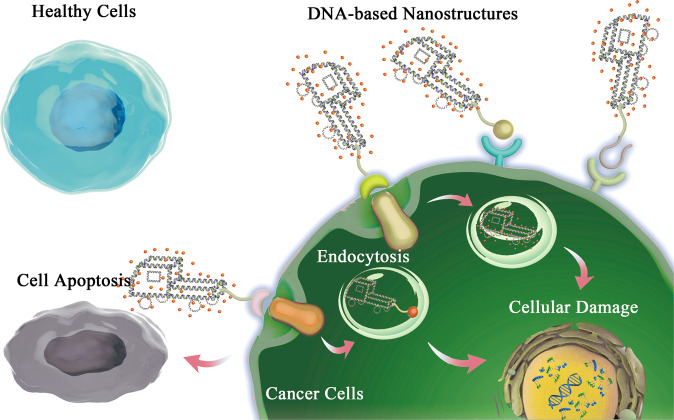
DNA-based nanostructures on tumor therapy by modification with drug, aptamer, or other functional ligands act various biological functions, which can bind to the target protein of cells. DNA-based nanostructures could distinguish cancer cells via ligand-receptor binding and permeate the membranes through endocytosis. The corresponding release of therapeutical agents would cause cellular damage and lead to apoptosis of cancer cells

Compared with conventional nanoscale drug carriers, such as gold nanoparticles and liposomes, DNA-based nanostructures are more suitable candidates characterized by high biocompatibility, structural diversity, low cytotoxicity, high accessibility, and capability of penetrating cell membranes without transfection.^412–414^ Owing to their nucleotide-composed backbones and flexible joints, DNA-based nanostructures possess high editability and modifiability. They can be built into arbitrary constructions like stacking building blocks.^38,415^ According to their intrinsic natures, multifunctional ligands can be integrated into frameworks through different ways, including conjugation, intercalation, encapsulation and loading.^416,417^ Thus, numerous assemblies of DNA nanoparticles were developed and further applied to multiple biomedical fields, especially in drug delivery (Fig. 3).^30,418^ In the following, diverse DNA nanoparticles applied in biomedicine are listed and illuminated (Table 2).

**Table 2:** DNA-based nanostructures on tumor therapy and cell imaging

Number	DNA-based nanostructures	Proteins or aptamers /target	Modification type	Payloads	Loading way	Application	References
1	Straight and twisted DNA tubes, DNA triangle	N/A	N/A	DOX	Intercalation	Cancer therapy	^424,425^
2	TDNs	FA/folate receptor	Click chemistry	DOX	Intercalation	Cancer therapy	^426^
3	DNA tube	AS1411/NCL	Linkage at the ends	Thrombin	conjugating through cross-linkers	NCL-overexpressed cancer therapy	^427^
4	DNA icosahedron	MUC1 aptamer /MUC1	Chemical bond	DOX	Intercalation	MUC-positive cancer therapy	^429^
5	DNA dendrimer	AS1411/NCL + MUC1 aptamer /MUC1 + ATP aptamer/ ATP	Chemical bonds	EPI	Intercalation	Cancer therapy	^430^
6	TDNs	HApt /HER2	Chemical bond	N/A	N/A	HER2-positive cancer therapy	^439^
7	TDNs	Affibody/HER2	Chemical bond	DOX	Intercalation	HER2-positive cancer therapy	^437^
8	TDNs	AS1411 /NCL	Chemical bond	5-FU	Chemical bond	NCL-overexpressed cancer therapy	^88^
9	TDNs	SL28 /VEGF-165 + FA /folate receptor	click chemistry	DOX	intercalation	Colorectal cancer therapy	^438^
10	TDNs	MUC1 aptamer /MUC1 + AS1411 /NCL	Extend from vertex + overhang on vertex	DOX	Intercalation	Cancer therapy and cell imaging	^449^
11	TDNs	AS1411 /NCL + GS24 /TRF	Chemical bond	TMZ	Load	Glioblastoma therapy	^450^
12	TDNs	AS1411/NCL + MUC1 aptamer/MUC1	chemical bond	_Ir_	Load	Glioma therapy	^452^
13	TDNs	N/A	N/A	MB	Interaction	Cancer therapy	^454^
14	DNA triangle	N/A	N/A	BMEPC	Interaction	Cancer therapy	^455^
15	TDNs	Nuclear localization peptide /nucleus	Chemical modification	ASOs	Disulfide linkage	Gene *c-raf* silence	^433^
16	X-Y-shaped DNA	Sgc8/ CCRF-CEM cancer cells	Linked with Y-shaped DNA strand	DOX	Intercalation	T cell acute lymphoblastic leukemia therapy and MDR	^462^
17	DNA nanotrain	Sgc8/ CCRF-CEM cancer cells or AS1411/NCL	Chimeric aptamer-trigger	DOX, EPI or daunorubicin	Intercalation	Targeted cancer theranostic	^463^
18	NFs	Sgc8c /PTK7	Integrated with template sequence	DOX	Intercalation	PTK7-overexpressed cancer therapy	^464^
19	DNA nanocircuit	Aptamer/cancer cells	Linked with an overhung catalyst sequence	Chlorin e6	Modified on ssDNA	Cancer therapy	^465^
20	DNA nanocentipede	Zy1/ hepatoma SMC-7721 cells	Streptavidin-based linkage	DOX	Intercalation	Hepatoma cells SMC-7721 targeted delivery	^466^
21	DNA nanorod	N/A	N/A	DOX	Intercalation	MDR	^108^
22	NFs	N/A	N/A	DOX	Intercalation	MDR	^484^
23	TDNs	N/A	N/A	DOX	Intercalation	MDR	^109^
24	DNA triangle and tube	N/A	N/A	DOX	Intercalation	MDR	^482^
25	TDNs	N/A	N/A	PTX	load	MDR	^107^
26	TDNs, square, pentagon-based pyramid and prism	PfLDH aptamer /PfLDH	Linked with ssDNA	N/A	N/A	Malaria diagnosis	^501^
27	TDNs	ANG /LPR-1	Click chemistry	N/A	N/A	Brain tumor imaging	^502^
28	TDNs	FAM and HE	Amido bond	N/A	N/A	H + and O^2^- detection	^504^

#### DNA polyhedron and origami

Since the first closed DNA polyhedron (poly-DNA) developed by Seeman et al., multifarious DNA-based predetermined motifs (tubes, buckyballs and prisms) were created.^419–421^ By means of DNA origami technique, the long scaffold ssDNA coupled with short and flexible staples provide more conceivements for different DNA motifs. For further developments and applications, poly-DNA was served as a nanocage to accommodate pharmaceutical molecules owing to its inherent spatiality.^416–418^ Extended sticky ends can also be designed for the attachment of additional aptamers, biotins or valuable peptides thanks to their modifiable external surface.^422^ With the modification of available ligands, it holds great promise in tumor targeted therapy.^423^

It is common to utilize DOX to form a drug-DNA nanocomplex. For instance, Hogberg et al. employed two types of DNA tubes (straight and twisted with different numbers) for carrying DOX and compared their carrying performance.^424^ Triangular DNA and tubular DNA of different forms were constructed as a vehicle to deliver DOX into cancer cells for effective cancer therapy.^425^ Folic acid (FA) coupled with DOX were also linked to TDNs,^426^ which indicating the excellent delivery abilities of TDNs.

A DNA rectangle bent into a hollow DNA tube, and the butt seam of which was fastened with AS1411 (a DNA aptamer).^427^ Its open state depended on the affinity of aptamer with the nucleolin targeting. With the structural separation, inner thrombin was exposed selectively to induce blood coagulation and cause cell death by vascular occlusion. Biocca et al. reported that truncated DNA octahedron (octa-DNA) could be recognized by low-density lipoprotein receptor-1 (LOX-1) because of its similar property with oxidized low-density lipoprotein (ox-LDL).^428^ It could thereby invade into LOX-1 related cells via substrate-receptor mechanism, which indicated the inherent targeting property of octa-DNA. Huang et al. proposed a unique DNA icosahedron (icosa-DNA) that was composed of six-pointed star skeleton.^429^ MUC1 aptamer was appended to its primary synthetic unit, and DOX was simultaneously embedded into double-stranded docking sites.^416^ This MUC1-DOX modified icosa-DNA could distinguish the MUC1-positive cells and achieve controllable release of DOX intracellularly.

As the non-stop development, multiple-bundle DNA-based nanostructures for different purposes appeared. An aptamer-Epirubicin (EPI) DNA dendrimer was designed by Khalil et al.^430^ Distinctively, more than two aptamers (AS1411, MUC1 and ATP aptamers) coupled with Epi were embraced in this bracket, equipping it with multitargetness and potent cytotoxicity.^423^

#### Applications of TDNs in drug delivery

Among multitude architectures, TDNs is of the greatest interest.^412,413,431–434^ Owing to its tripod-shaped framework, excellent structural stability was conferred so that it could resist deformation under enzymatic digestion. As for the synthesis of TDNs, several synthetic schemes were put forward, and TDNs of different sizes were successfully produced.^169,435,436^ These products were examined with potential to remedy many disorders. In previous studies, aptamers (such as AS1411, anti-HER2 aptamer (HApt), and SL2B), antibodies and proteins (affibody, FA) were conjugated with TDNs for cancer cell recognition and inhibition.^437–439^ Coupled with the loading of therapeutical agents like 5-fluoroutacil (5-FU), DOX, paclitaxel (PTX), the products were supposed to selectively enter cancer cells and exert potent antitumor effects.^88,107,426^

As a proof of concept, HApt was linked with TDNs to anchor HER2-overexpressed cancer cells.^439^ HER2 is a member of transmembrane proteins, and a hub for signal transduction pathways among cancer cells. Its overexpression is associated with malignancy and the poor prognosis of breast cancer.^440^ According to previous work, HApt could be specifically chosen to bond with HER2 and supress its expression.^441,442^ With this in mind, HApt-TDNs was prepared to specifically combine with HER2 and then transfer it into lysosome for the following degradation. This behavior consequently contributed to cell apoptosis and inhibit tumorigenic growth. Compared to simple HApt, the stability of this nanocomplex was considerably improved and response time was prolonged. It might stand for a new perspective in breast cancer therapy. In addition, a tailored affibody molecule was built to mimic the structure of monoclonal antibody, which was confirmed with specificity to target HER2.^437^ With the additional intercalation of DOX, this DNA-affibody-drug chimera showed excellent specificity and inhibition to HER2-overexpressed cancer cells.

With regards to aptamers, G-quadruplex aptamer (AS1411) was the representative one that was systermatically investigated. It was equipped with both targetness and pleiotropic antiproliferation through its combination with targeting receptor-nucleolin (NCL).^443,444^ It could be directly attached to the vertice of poly-DNA or linked with the ssDNA to form an overhang at the edge of 3D structure.^445^ These AS1411-modified poly-DNA could act as an effective drug carrier or a nanomedicine with targeting ability for therapy.^100^ Studies have also been concentrating on the structural upgrading of TDNs.^446–448^ AS1411 and 5-FU were simultaneously conjugated to TDNs to establish a splendid nanomedicine that specifically inhibit the cell viability of NCL-overexpressed cancers.^88^

SL28 is a VEGF-165-related DNA aptamer. Its dimerization with VEGF-165 plays an important role in angiogenesis in vivo. Zhao et al. presented a dual-targeting nanosystem for SL28 and FA, the cooperation of which could obviously improve targeting ability of nanosystem towards colon cancer cells.^438^ With a follow-up loading of DOX, the synergic antitumor effect was obtained. Liu et al.^449^ united MUC1-probe and AS1411 with TDNs, as well as the payload of DOX. The selective MUC1 aptamer-protein binding induced an unwinding of quenched sequence and fluorescence recovery to differentiate MUC1-positive cancer cells. The follow-up AS1411-nucleolin bound caused its translocation into nucleus and DOX release intranuclearly. This superior combination carved a path to cancer theranostics. Furthermore, temozolomide (TMZ) loaded TDNs along with two DNA aptamers (AS1411 and GS24) for glioblastoma treatment was reported.^450^ GS24 was reported to be a transferrin (TRF) aptamer of 64 nt in length.^451^ Owing to its unique spatial structure, TRF on cerebrovascular endothelial cells of BALB/c nude mice can seize and combine with GS24, and thereby pass through cerebrovascular barrier. Specifically, after injection with this nanointegrate, the function route was as follows: firstly, it moved across the blood-brain barrier (BBB) of mouse and permeated into brain parenchyma via receptor-mediated transcytosis; secondly, the assistance of AS1411 enabled it to enter the cell nucleus, and then offloaded TMZ for its lethality towards tumor cells, which could alleviate the resistance and myelosuppression of TMZ at the same time.

Other elements including metal complex and photosensitizer were also added as adjuvants to substantially enhance the medical effects of TDNs. Tian et al.^452^ employed a dual aptamer-tethered TDNs for carrying metal compounds. A representative platinum agent-[Ir(ppy)_2_phen]^+^PF_6_ (Ir) was loaded onto MUC1- and AS1411-modified TDNs. It anchored glioma cells to destroy their vascular mimicry processes and cause cell death through mitochondrial and ROS pathways, which contributes significantly to glioma therapy.

In addition to routine drug treatment, photodynamic therapy (PDT) is a minimally invasive means, which is frequently used in tumor therapy. Photosensitizers including porphyrinoid and cyanines can be activated under irradiation, thereby produced excess ROS and ultimately drove cancer cells to necrose.^453^ When they are attached to or loaded on TDNs, their disadvantages such as low stability, poor solubility and tissue penetration were greatly minimized. Methylene blue (MB)^454^ and BMEPC,^455^ which are commonly used as imagine probes and methemoglobinemia agents, can also interact with TDNs and DNA origami. Subsequently, when the preprocessed tumor tissues were exposed to light, a satisfying cytotoxicity against tumor cells would be observed.

Other than chemical agents, the delivery of antisense oligonucleotides (ASOs), proteins and RNA are also spotlighted. Dual-bundle TDNs with targeting peptides and ASOs were reported to effectively deliver ASOs into nucleus of tumor cells.^433^ The increased release of ASOs could lead to an enhanced cytotoxicity and the knockdown of proto-oncogene *c-raf*. Studies regarding small interfering RNA (siRNA) were also reported.^431,456,457^ Similar DNA nanoparticles with cancer-targeting peptides were utilized to accurately transport siRNA into tumor cells and trigger related genes silencing. The low expression of particular genes is the main inducement of cell inhibition.

In addition to the above-mentioned, TDNs were also proved to affect various pathological courses. Lin et al. reported that TDNs could modulate the M1 polarization of macrophages into an appropriate level, avoiding an exaggerated inflammatory response.^458^ It also exhibited antioxidative and anti-inflammatory potentials through suppressing mitogen-activated protein kinases (MAPK, including JNK1/2/3, ERK1/2, and p38 family) and upregulating the expression level of *HO-1*, which is associated with anti-inflammation, anti-apoptosis and antioxidation.

In the microbiological researches, TDNs have sparkled as a component of innovative antimicrobial agents. With the advent of superbugs, bacterial resistance caused by antibiotics abuse has become so acute that effective measures should be immediately taken to prevent the deterioration of this condition. An antisense peptide nucleotide (asPNA) with high stability and affinity was proposed to inhibit the transcription of an intended bacterial gene (*ftsZ*).^459^ Its encoded protein (FtsZ) was confirmed to be involved in bacterial cell division and has been used as a validated target in bacterial prevention. Lin et al. incorporated asPNA with TDNs to deliver it into methicillin-resistant *Staphylococcus aureus (MRSA)*, interfere the expression of *ftsZ*, and subsequently result in bacterial growth inhibition. Although humans are collective organisms with microbes, the colonization of bacterial biofilm is a decisive factor of chronic inflammation, which is the main culprit of critical illnesses in essential organs. Microbial communities form compact biofilms, along with exopolysaccharides (EPS) and other extracellular matrix, to protect themselves from antibiotics. Therefore, the most fundamental method to diminish the vitality of bacteria is to prevent the initial formation of bacterial biofilms. The same study group designed an ASOs on the basis of VicK protein binding domains to regulate the secretion of EPS for early prevention against biofilm formation.^460^ It could specifically target *gtfBCD* (from glucosyltransferase gene family)*, gbpB*, and *ftf* genes, which encoded a series of *Streptococcus mutans* biofilm formation proteins, a glucan-binding and an adhesion-associated protein, respectively. Similarly, ASOs were carried by TDNs to degrade the expression of targeted genes and availably block EPS production, which were both prerequisites for initial stage of biofilm.

#### DNA intelligence nanodevice

Along with the progress in DNA nanotechnology, deoxynucleotides were concatenated, folded, and assembled into a more intricate, multifunctional and intelligent nanodevice.^461^ Primitively, Tan et al. created X-Y-shaped DNA nano-assembly^462^ and aptamer modified DNA nano-train.^463^ For the former, three branches of Y-shaped DNA units were developed to link or intercalate with aptamers, drugs or oligonucleotides, and the chimera then jointed in the X-shaped central bonder through predesigned supplementary arms. After compact crosslinking of these X-Y-shaped building blocks, a simple nano-assembly with targeting and multiple drug resistance (MDR) reversion ability could be obtained. The latter one was composed of two hairpin-patterned DNA sequences (M1, M2) and an aptamer trigger. The hybridization of M1 and M2 was initiated by the mixture of chimeric trigger, resulting in a nano-train with a chimeric aptamer locomotive and many chambers for drug loading. The same group intensively studied a noncanonical DNA nanoflower (NFs) without following Watson–Crick.^464^ It was self-assembled from DNA concatemers generated by rolling circle replication and the building template could be tailored with the attachment of functional moieties for biotherapy and bioimaging, etc. DOX and aptamer sgc8c were embedded and incorporated into NFs to recognize PTK-overexpressed cells and exert therapeutic effects. The optical labels could be similarly applied for fluorescent imaging. Moreover, several novel DNA nanocircuits were also established.^465^ The trigger sequence linked with the aptamer could precisely activate a series of related cascade reactions in target cells to eventually produce therapeutic effects.

Xu et al. fabricated a DNA nanocomplex with a trunk and several legs, resembling a centipede.^466^ Two hairpin sequences constituted a DNA scaffold through hybridization chain reaction (HCR) to form a long DNA trunk, providing abundant docking sites for pharmaceutical molecules. Aptamers such as Zy1 were combined with the trunk-like couples of legs via streptavidin-biotin affinity. It could grasp hepatoma cells SMC-7721 and release the payload to influence the cell activity. Theoretically, this nanocentipede could be further used in multivalent drug delivery.

Moreover, smarter 3D architectonics designed for drug containers like DNA boxes were set up with movable switches.^145^ Their hollow constructions provide sufficient spaces for functional cargoes. Contents could be reversibly exposed or manageably released under genetic, optical or many other biochemical triggers.^467^ Additionally, more sophisticated lock-and-key systems were fabricated to control the accessibility of cargoes or for controlled reaction rate as required.^468^ Their structures were also refined to the greatest extent so that they could readily pass through the biological barriers in vivo.

For DNA nanodevices illustrated above, the appended molecules were mostly replaceable, revealing that they were superior to drug carriers with broad feasibility. However, the microstructures of the combination between DNA nanomaterials and different drugs cannot the clear observed.

#### DNA nanohydrogels

As for particle size, apart from individual nanoscale structures, micron-sized DNA polymers were constructed via liquid phase crystallization and dense packing of dsDNAs instead of Watson–Crick base-pairing.^469,470^ Thus, they exhibit excellent monodispersity and biocompatibility. Tan et al. proposed a multi-module DNA nanohydrogel with one linker and two functional monomers.^471^ The size could be adjusted by controlling the ratio of building units. Elements including aptamers and genes could be incorporated to build an efficient nanomedicine. Hitherto, not just linear as nanowires, other devisable and rigorous nanostructures have been obtained. They were applied extensively in both biosensing and biomedicine, and would be further utilized in biological nanotechnology.

#### Switchable DNA nanomachine

In order to meet the growing demands, mechanically controlled DNA nanomachines with operational strands that could be switched on and off by active domains emerged.^472–474^ For instance, complementary fuel strands were designed to initiate DNA tweezers.^475^ Their hybridization and removal enable these tweezer-like machines to open and close reversibly. In addition, the structural transformation could be triggered by following fuses: changes of pH, light excitation, binding with specific sequences or incorporating with proteins, aptamers or other functional ligands. A pH-dependent DNA origami plier with two levers connected at a fulcrum was observed to transform according to pH value.^476^ Its X-shaped open state could turn into parallel or antiparallel closed form in response to acidic conditions.

At present, a new concept-DNA nanorobot is emerging. It refers to an array of highly intellectual nanodevices. They could be equipped with multiple functions through precision assembly. The mode of action was controlled by organized switching system, which includes but was not limited to simple on-off setting, and the logic gates (AND, NOT, OR, etc.) could also be set up.^477,478^ Thubagere et al.^43^ devised an autonomous DNA robot for cargo sorting. Unlike previous ones that walked following a prescribed route, it could walk towards different directions randomly without energy supply for automatic cargo detection and classification. Three building blocks were designedly composable to select various cargoes at uncertain locations and classify them into two groups. This sorting task could be carried out on the surface of a 2D DNA origami sheet. In more sophisticated scenarios, several robots could be assigned to work cooperatively. When it comes to other molecules, such as antibodies, proteins or nanoparticles, promising applications including molecular computation and biomedical diagnosis can be explored.

### Multi-drug resistance (MDR) reversion

DNA-based nanostructures can not only achieve effective delivery of chemical agents to tumor sites, but also reverse multi-drug resistance (MDR) in cancer cells. MDR, either inherent or acquired, has always been a tough challenge in drug development and utilization. To overcome it, multifarious drug combination strategies are proposed and different drugs are formulated, but their effects are not that satisfactory.^479^ The causes of MDR are extremely complex, including DNA repair, decreased drug flow and detoxification. According to previous studies, the most common reason for this is the excessive efflux pumps, which can eject drug and result in ineffective.^479,480^ Taken together, the fundamental way to bypass this efflux-pump mediated MDR is to improve drug concentration in the pathological areas.

DNA-based targeted drug delivery system has become a focus worldwide because of its ability to increase drug accumulation specifically in cancer cells via enhanced permeability and retention effects (EPR).^481^ It proved that DNA nano-systems could enter cells through caveolin- and clathrin-mediated endocytosis, independent of efflux pump pathway.^413^ Therefore, their high delivery efficiency can be expected to reverse obstinate MDR observed in most drug tests.

Conventional agents such as DOX, PTX, 5-FU have been confirmed to elicit MDR in tumor cells during their long-term use. Investigators have been striving for a more optimized program-various types of DNA nanoparticles that were applied for DOX resistance.^481–483^ As a consequence, DNA origami was reported to show better performance in delivering effective levels of DOX than poly-DNA owing to its higher density of DOX in more abundant docking sites. DOX loaded rod-like,^108^ X-Y-shaped DNA origami and an apt-NFs nano-assembly^484^ were fabricated for human leukemia/MDR cells, respectively. A DOX loaded TDNs was found to inhibit MCF-7/MDR cells activities.^109^ MDR reversion in these cases were achieved through efflux evasion, which directly enhanced local drug concentration, and thus, extended effective action time and reduced the general toxic effects. Additionally, Ding et al. constructed 2D (DNA triangle) and 3D (DNA tube)^482^ nanostructures to carry DOX. Both of them could improve drug sensitivity of DOX/MDR human breast cancer cells through efflux pathway. They were verified to be able to prompt drug molecules to disperse into active sites through elevating lysosomal pH.

PTX loaded TDNs (PTX-TDNs) presented by Lin’s group was surprisingly valuable for treatment of PTX/MDR non-small cell lung cancer.^107^ It exerted cytotoxicity towards PTX/MDR cancer cells through downregulating the expression of P-glycoprotein (P-gp), a membranal transporter associated with drug efflux. In other words, PTX-TDNs could increase intracellular excretion of drugs in MDR cells, resulting in potent microtubule polymerization and cell apoptosis.

### Immunostimulatory effect

**Fig. 4:**
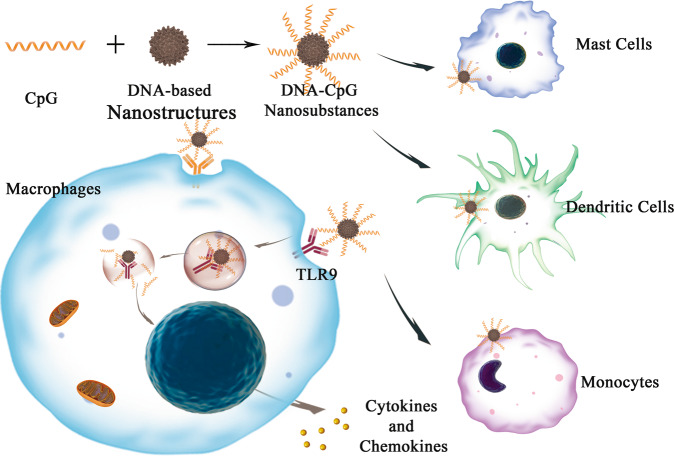
DNA-based nanostructures on immunostimulatory reactions. CpG-modified DNA-based nanostructures could enter immune cells, including macrophages, mast, dendritic and monocyte cells through integrating with TLR9, which subsequently induce cascade reactions and promote the secretion of remarkable cytokines and chemokines

Similar to aptamers and drugs, ligands with immunostimulatory (e.g., CpG), or catalytic activities (e.g., enzyme) can be appended to DNA frameworks.^485^ Since the basic component of DNA nanoparticles is deoxyribonucleic acid, they exhibit low immunogenicity and their internalization would not intensify immunoreaction. Unmethylated CpG oligodeoxynucleotides can be recognized by mammalian immune system because of its existence in bacterial or viral genome.^486,487^ It can specifically combine with Toll-like receptor 9 (TLR9), and activate it allosterically, which subsequently induce cascade reactions and promote the secretion of remarkable cytokines and chemokines, including interleukin-12 (IL-12) and tumor necrosis factor-α (TNF-α).^488,489^ Thereout, DNA-CpG nanosubstance was conceived an immunomodulating nanomaterial (Fig. 4).

DNA nanotubes^490,491^ of different dimensions and DNA dendrimers^492,493^ with CpG were reported in previous studies. Fan’s team attached CpG to four ssDNA building units and prepared a CpG-bearing TDNs.^494^ Variform DNA nanoribbons based on rolling circle amplification (RCA) technique were proposed by the same group as well.^495^

In general, macrophages along with dendritic, monocytes and mast cells are of great significance in maintaining immune homeostasis and play an important role not only in autoimmunity, but also in inflammatory diseases, metabolic disorders, neoplasia, anaphylaxis and wound healing. CpG-modified DNA-based nanostructures could enter immune cells, including macrophage RAW264.7 cells noninvasively and efficiently, and keep intact in their endosomes, whereupon trigger enhanced immune responses.^496^ Through this activation process, they were expected an alternative to surmount tumor-associated immunodepression and regulate essential pathological reactions. Afterwards, CpG was respectively linked to ssDNA, dsDNA, Y-shaped DNA (Y-DNA), and three types of dendritic DNA (DD-DNA).^492,497^ In the literature, Y-DNA with three dendritic branches served as a single element in DD-DNA’s building-up process. One Y-DNA was ligated to three other duplicates to obtain a primary DD-DNA (D_1_). One D_1_ was thereby ligated to another six Y-DNA to form a secondary DD-DNA (D_2_). By that analogy, tertiary DD-DNA (D_3_) was synthesized. It was noteworthy that individual Y-DNA, D_1_, D_2_ and D_3_ without CpG were substantiated to have intrinsic immune activation properties, and there was an increase with the growth of branch number, maybe partially due to their increased uptake by macrophages. Then, CpG were positioned on the outermost layer of each structures. In other words, D_1_ (CpG), D_2_ (CpG) and D_3_ (CpG) were decorated with 6, 12 and 24 CpG sequences, respectively. Detailed experiments revealed that the secretion levels of TNF-α and IL-6 were strikingly increased in DD-DNA (CpG) and Y-DNA (CpG), compared to ssDNA (CpG), dsDNA (CpG) and simple mixture of several CpG sequences. However, the increasing trends were not proportional but much higher than the enhanced immunoreaction triggered by non-CpG structures. It was indicated that the immunostimulation of CpG motifs could be greatly amplified by the structural characteristics of Y-shaped DNA dendrimer. A similar profile was detected in the comparison of Y-DNA modified with 1 and 3 CpG motifs. Thus, more CpG motifs on one architecture might stimulate stronger immune response. This finding was then extensively applied to inhibit the growth of tumor cells. After adding Y-DNA (CpG)-treated RAW264.7 cells, a significant decrease was observed on the number of murine melanoma cells. To clarify the relationship between structural features and biological properties, the same team established some other polypod DNA (p-DNA).^498^ They used 3 to 12 oligodeoxynucleotides of different lengths to generate tri-, tetra- to dodecapods DNA of different sizes, and CpG was attached in a similar way. Through rigorous examinations, it was discovered that p-DNA with more pods entered RAW264.7 cells more easily. The release levels of TNF-α and IL-6 showed the same trend, and an increasing order was found in tri-, tetra-, penta-, hexa- and octapods DNA, which symbolized a stronger activation of immune response. These results provided definite evidence that the growing number of pods had an impact on activating the level of immunostimulation.

To the best of our knowledge, the invasion of foreign substances would cause varying degree of host immune defense. In general, it manifests as an excess secretion and accumulation of inflammatory cytokines, which is the origin of systemic complications. To explore the immunological role of TDNs, previous studies established lipopolysaccharides (LPS)-induced inflammatory cell model to act RAW264.7 cell line.^458^ After retreatment with TDNs, the production and the expression of nitric oxide (NO) and inducible NO synthase (iNOS, closely related to NO production) were decreased and downregulated to regulate NO within a normal concentration. Herein, it could be inferred that TDNs is an outstanding biomaterial without immune rejection. Based on this, Chang et al. utilized TDNs as an antigen platform for vaccine.^432^ As CpG conventionally serves as an adjuvant in vaccine for immunotherapy, Chang et al. assembled an antigen-streptavidin (STV), CpG and TDNs into a vaccine complex (SCT). Both in vitro and in vivo studies showed that STV and CpG could be more easily internalized by antigen-presenting cells due to the application of TDNs delivery system. Then, more T cells were activated, facilitating the differentiation of STV-related B cells and the production of relevant antibodies. Importantly, the 3D architecture of SCT could render a close proximity of adjuvant and antigen, which highly augmented immunogenicity of the vaccine. Given results above, DNA-based nanosystems maintained an impressive immunostimulation potential that might be further utilitized in immunotherapy and vaccine development.

### Biosensing, biodetection, and diagnosis

**Fig. 5:**
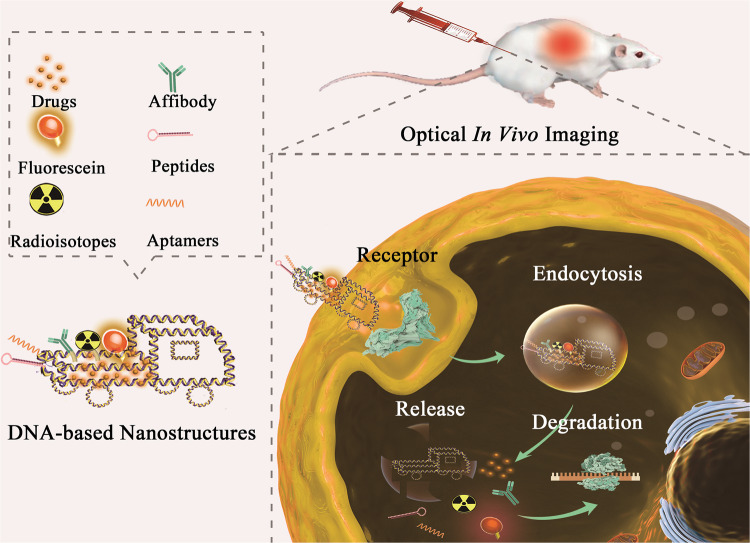
DNA-based nanostructures on bioimaging and diagnosis. Targeted ligands (e.g., affibody, peptides and aptamers) and cargoes (e.g., drugs, fluorescein and radioisotopes) were simultaneously attached to DNA-based nanostructures. After specific combination with biomarkers, the integrates entered target cells and released fluorescent labels to sensitively detect the diseased regions

Early diagnosis of diseases is of great importance in developing timely and effective treatment stragety to prevent deterioration and cure illness.^499,500^ The traces of intracellular molecules, proteins or nucleic acids have great benefits in disease prevention, diagnosis and therapy. Conventionally, experimental approaches such as western blotting to assay their expressions are time-consuming and laborious. Micromolecular probes that can achieve real-time detection and dynamic imaging are preferable. A robust probe delivery vehicle is on demand to conquer the instability of simple probes. More recently, DNA-based nanostructures show extraordinary promise in molecule detection and biomedical imaging (Fig. 5).^413^

Generally, aptamer-based DNA diagnostic nanodevices were widely explored. TDNs, square, pentagon-based pyramids and prisms were incorporated with *Plasmodium falciparum* lactate dehydrogenase (PfLDH) aptamer and fluorescent labels to distinguish malaria biomarker- PfLDH from human lactate dehydrogenase.^501^ Recent progress has been made that angiopep-2 (ANG) linked TDNs (ANG-TDNs) was a potential imaging medium for brain tumors. Owing to the high affinity of ANG with low-density lipoprotein receptor protein-1 (LPR-1) on brain endothelial and glioma cells, ANG-TDNs was able to cross BBB and recognize cancer cells,^502^ and combined ues of luminescent dyes could specifically label brain tumor regions.

In Argonaute2 (Ago2) detection, Zhang et al.^503^ designed a switchable nanoprobe using TDNs as a scaffold. An RNA-hairpin sequence was linked to the terminal of one strand, and a photoinduced electron transfer pair (PET) was attached to the terminal of the complementary strand. The vicinity of them caused a fluorescent quenching, while the emergency of Ago2 and its corresponding siRNA could restore its fluorescence. Under conditions of inflammation, neurodegeneration and most diseases, abnormalities of hydrogen ions (H^+ ^) and superoxide anion (O_2_^-^) can be discovered. Li et al.^504^ incorporated hydroethidine (HE) and fluorescein (FAM) with the vertex of TDNs, the intensity of fluorescence would change in response to pH and O_2_^−.^ Hence, a sensitive nanoprobe monitoring microenvironmental changes emerged for future disease diagnosis. The reason why TDNs was frequently employed as a bioimaging vehicle is that its rigid skeleton renders a fixed distance between fluorescent labels. It can prevent attached probes from self-quenching, and control their fluorescence recovery. In some cases, aptamer strand acts as not only a molecular recognition probe but also an activatable initiator. An electrochemical TDNs without additional reagent operated as follows:^505^ Once the target was captured by strand a and bounded with it, strand b on another vertex could pull them down through hybridization; the consequential collision of the terminal probe with electrode surface was induced, and eventually caused electron transfer. This linkage mechanism can be achieved in living animals, and thus expected to be applied in point-of care testing.

In practice, bioimaging-related agents (e.g., ^99m^Tc) are all candidates that could be integrated with TDNs. Indeed, multiple probes can be simultaneously functionalized for a more sensitive imaging nanodevice. Jiang et al.^506^ incorporated FA, radioisotope and ordinary label on TDNs with the linkage of multiple arms. A multimodality probe that can be observable under both near-infrared (NIR) and single-photon emission computed tomography (SPECT) was achieved.

Beyond that, metallics and fluorophores were also commonly used in bioimaging and biosensing. A novel DNA nanoprobe containing AuNP, three Cyanine-5 (Cy5) and custom telomerase strand primer (TSP) was integrated for telomerase monitoring. Its fluorescence was quenched by fluorescence resonance energy transfer (FRET)-induced AuNP at an original state, which was regarded as “off”. After catching the signal of telomerase, hairpin structural TSP linked with AuNP would unfold, leading to the fluorescence recovery of Cy5 (“on” state). The sensitive detection of telomerase in cancer cells was a referable forebode in early diagnosis.^507^

In clinical, analysis of lymphonodi is essential in estimating oncology progress. Sentinel lymph nodes, the primary ones which malignant cells invade, become the emphasis of auxiliary examinations. However, case investigation reveals that patients with aggressive tumors like melanoma are susceptible to metastases after sentinel lymph nodes biopsy. Alternative noninvasive means are necessary. Dye-labeled TDNs with high in vivo stability, long circulation and great visualization was prepared, not only for lymph node imaging but also for tissues evaluation.^434^

### Cell surface engineering

The cell surface membrane is the primary mediator for interactions of the cell-cell and the cell-microenvironment.^508^ Recently, the DNA nanomaterials were also investigated to modify the cell surface membrane for different aims, such as targeting, drug delivery, and enhanced interactions of cell-cell and cell-microenvironment.^509^ Gartner et al.^510^ have demonstrated that hybridization of complementary DNA sequences enabled the assembly of multicellular structures with defined cell-cell contacts. Akbari et al.^511^ also reported a DNA nanoplatform that could act as a membrane-bound breadboard (MBB) with nano-scale. This work developed a foundation to apply DNA nanomaterials as membrane engineering technologies, which could mimic and direct complex biomedical processes on the cell surface of different cells in tissues. Some DNA nanomaterials would be investigated for the cell surface engineering with some targeted aims, such as drug delivery and gene transduction. Furthermore, some DNA nanomaterials could combine with the proteins of cell membrane, and then could be internalizated by cells.Some studies have been deomenstrated that some DNA nanomaterials could transfer the cell membrane into the biomimic materials, which could act some functions in the palsma of the living cells.

In the future studies, the membrane-bound DNA origami structures could enable programed assembly within the membrane to mimic functional biomolecular complexes, such as an immune synapse, especially given the wide use of DNA origami to template or organize proteins.^512^

## Summary and perspective

In recent decades, DNA-based nanostructures have become one of the most attractive focuses because of their structural diversity and scientific values. As a basic component of living entities, DNA is endowed with excellent biocompatibility. Its 3D double-helix structure and small molecular weight confer variability and nanosized magnitude. As mentioned above, DNA nanoparticles can be assembled into arbitrary shapes including 2D triangle, rectangle, 3D polyhedron, liquid nanohydrogel, smarter nanocontainer and biomimetic constructions. More fancy structures might be available in the future.

More importantly, DNA nanoparticles have showed influences on cell biological activities, especially on cell proliferation. Accordingly, their roles in tissue regeneration were broadly investigated in tissues including bone, cartilage, blood vessel, nerve, skeletal and cardiac muscles, skin and corneal systems. They also attract tremendous attentions when they serve as nanocarriers. Compared with traditional nanoparticles, they are more variform for the following reasons: (1) flexible joints because every strand of DNA-based nanostructures can be concatenated or linked with an extended arm; (2) abundant binding sites, as the assembly of DNA frameworks provides a hollow internal space for drug molecules; (3) negative charge, so that positive-charged substances can be integrated with them under electrostatic attraction. In this context, studies started applying them as nanoscale vehicles to transport various drugs into cellular focal sites for a desired efficacy. Therapeutical moieties with distinctive natures were chosen to incorporate with DNA nanoparticles. Aptamers (AS1411, MUC1, HApt, etc.) and antineoplastics (DOX, PTX, 5-FU, etc.) modified nanostructures were the most attractive. Their capabilities of antitumor effect, MDR reversion and photodynamic promotion were discovered. Then, the notion of multi-modality nanosystems were put forward to enhance their targeting performance. In addition, through the combination with immune agents (e.g., CpG) and fluorescent labels (e.g., FAM), DNA nanoparticles were also of great significance in immunostimulating, biosensing and bioimaging. More advanced nanotools with logic gates or structural switches were developed for further accurate administration.

Although the biological applications of various DNA nanostructures were comprehensive and extensive, there remain some limitations to overcome, such as, the interaction mechanisms between DNA nanomaterials and cells, the short blood circulation time, and poor aggregation in target tissues. The cellular internalization processes of various DNA nanomaterials are the prerequisites for biological applications. For example, the TDNs have been widely investigated in bioimaging, drug delivery, targeted delivery of functional DNA or RNA sequence, and biocatalysis.^502,513–515^ Unlike ssDNA with poor cellular uptake properties, previous studies reported that TDNs could be internalized by cells without the assistance of transfection agents.^516–518^ In the recent study, researcher combined drug affinity responsive target stability (DARTS) with liquid chromatography/tandem mass spectrometry (LC-MS/MS) techniques to explore the endocytosis process of TDNs. The study reported that the endocytosis of TDNs were related to caveolin-1 (CAV1) and micropinocytosis-related protein sorting nexin5 (SNX5). However, the cellular internalization processes of other DNA nanomaterials were not clear still now, which need more explorations.

For the nature of DNA nanomaterials, different DNA nanomaterials encounter the challenges of poor blood circulation time and poor aggregation in target tissues. Some previous studies have demonstrated that the DNA nanomaterials (TDNs, HApt-TDNs, and DNA aptamer, etc) could circulate in body no >1 h, which were quickly eliminated by different cells and organs (such as macrophages, liver, and kidney) before DNA nanomaterials arriving at target tissues.^519^ Therefore, some other bionic materials were investigated to prolong the blood circulation time, such as liposomes and cell membranes.^520^ Some previous studies have reported various DNA-coated liposomes for drug delivery.^520–522^ In a recent report, Han et al.^526^ found that tannic acid (TA) could mediate the co-assembly of branched-DNA/RNA and A549 lung cancer cell membrane-camouflaged to form nanocomplex (nanocomplex@A549m), which could strengthen the affinity between TA and nucleic acids/membrane proteins. The objectives of nanocomplex@A549m with the application of A549 lung cancer cell membrane-camouflaged were to prolong the blood circulation and reduce macrophage clearance. Moreover, the nanocomplex@A549m could effectively bind to target cells. From this study, we think that the cell membrane-camouflaged method (stem cells, cancer cells, blood cells, and exosomes, etc) might inspire other researchers to develop more DNA-based nanocomplex@cell-membrance materials.^520,523^ In further studies, DNA nanomaterials might be combine with other materials for more applications, such as hydrogel,^524^ nanozyme,^525^ and bimimic cell membrane nanomaterials.^526^

In conclusion, due to their structural flexibility, multiform customized DNA-based nanostructures were created. Some innovative creations discussed in this review provided valuable directions for the development of DNA-based nanostructures in biomedicine. Regarding the biological performance, acquired nanostructures were explored in tissue engineering and regeneration. As a high-efficient drug delivery system, they could be used as assembly templates to generate agent-modified nanodevices that execute rationally designed behaviors, including tumor therapy, MDR reversion, immunotherapy and biosensing. While studies and analyses were mostly conducted in vitro and the biological effects in different aspects are encouraging, in vivo studies are critical and needed to confirm the observed effects, assess the side effects and make the comprehensive evaluation before achieving the ultimate clinical application of DNA-based nanostructures.
